# Modeling the Function of TATA Box Binding Protein in Transcriptional Changes Induced by HIV-1 Tat in Innate Immune Cells and the Effect of Methamphetamine Exposure

**DOI:** 10.3389/fimmu.2018.03110

**Published:** 2019-02-04

**Authors:** Ryan Tjitro, Lee A. Campbell, Liana Basova, Jessica Johnson, Julia A. Najera, Alexander Lindsey, Maria Cecilia Garibaldi Marcondes

**Affiliations:** ^1^Department of Neurosciences, The Scripps Research Institute, La Jolla, CA, United States; ^2^LAC Intramural Research Program, National Institute on Drug Abuse, Baltimore, MD, United States; ^3^San Diego Biomedical Research Institute, San Diego, CA, United States

**Keywords:** HIV, Tat, Methamphetamine, TATA-box, TATA-box binding peptide, Crispr/Cas9, macrophages, inflammation

## Abstract

Innate immune cells are targets of HIV-1 infection in the Central Nervous System (CNS), generating neurological deficits. Infected individuals with substance use disorders as co-morbidities, are more likely to have aggravated neurological disorders, higher CNS viral load and inflammation. Methamphetamine (Meth) is an addictive stimulant drug, commonly among HIV+ individuals. The molecular basis of HIV direct effects and its interactions with Meth in host response, at the gene promoter level, are not well understood. The main HIV-1 peptide acting on transcription is the transactivator of transcription (Tat), which promotes replication by recruiting a Tata-box binding protein (TBP) to the virus long-terminal repeat (LTR). We tested the hypothesis that Tat can stimulate host gene expression through its ability to increase TBP, and thus promoting its binding to promoters that bear Tata-box binding motifs. Genes with Tata-box domains are mainly inducible, early response, and involved in inflammation, regulation and metabolism, relevant in HIV pathogenesis. We also tested whether Tat and Meth interact to trigger the expression of Tata-box bearing genes. The THP1 macrophage cell line is a well characterized innate immune cell system for studying signal transduction in inflammation. These cells are responsive to Tat, as well as to Meth, by recruiting RNA Polymerase (RNA Pol) to inflammatory gene promoters, within 15 min of stimulation (1). THP-1 cells, including their genetically engineered derivatives, represent valuable tools for investigating monocyte structure and function in both health and disease, as a consistent system (2). When differentiated, they mimic several aspects of the response of macrophages, and innate immune cells that are the main HIV-1 targets within the Central Nervous System (CNS). THP1 cells have been used to characterize the impact of Meth and resulting neurotransmitters on HIV entry (1), mimicking the CNS micro-environment. Integrative consensus sequence analysis in genes with enriched RNA Pol, revealed that TBP was a major transcription factor in Tat stimulation, while the co-incubation with Meth shifted usage to a distinct and diversified pattern. For validating these findings, we engineered a THP1 clone to be deficient in the expression of all major TBP splice variants, and tested its response to Tat stimulation, in the presence or absence of Meth. Transcriptional patterns in TBP-sufficient and deficient clones confirmed TBP as a dominant transcription factor in Tat stimulation, capable of inducing genes with no constitutive expression. However, in the presence of Meth, TBP was no longer necessary to activate the same genes, suggesting promoter plasticity. These findings demonstrate TBP as mechanism of host-response activation by HIV-1 Tat, and suggest that promoter plasticity is a challenge imposed by co-morbid factors such as stimulant drug addiction. This may be one mechanism responsible for limited efficacy of therapeutic approaches in HIV+ Meth abusers.

## Introduction

The infection with the Human Immunodeficiency Virus (HIV) triggers inflammatory pathogeneses in several tissues (3), including in the Central Nervous System (CNS) (4). Inflammation in the brain and cognitive deficits can be significantly aggravated upon the co-morbid use of addictive substances, such as Methamphetamine (Meth), which impair the success of antiretroviral therapies and increase viral load specifically in the brain (5, 6). The Meth-induced aggravation of HIV—induced CNS deficits, inflammatory response, and brain viral load, have been replicated in non-human primate models of neuroHIV (5, 7, 8). Of the HIV-1 peptides, the trans-activator of transcription (Tat) is the only one that is secreted across the plasma membrane (9–12), affecting neighboring cell types. *In vivo*, HIV-Tat is linked to impaired learning and memory, and gray matter deficits (13, 14), suggesting its involvement in HIV-associated neurological disorders (HAND). In a mouse model of neuroHIV, the induction of Tat in CNS glial cells enhances behaviors associated with Meth exposure, while contributing to changes in microglial activation and in the expression of neurotransmitter receptors (15).

In the brain, innate immune cells, microglia and macrophages, are targets of HIV infection. The main role of the HIV Tat peptide is maintaining viral transcription by activating the viral promoter, located within the 5′ long terminal repeat (LTR) of the integrated viral genome. In that process, the virus gains the ability to hijack the host cell RNA polymerase II (RNA Pol II) machinery for initiating viral transcription. In previous experiments, we observed that the SIV infection in rhesus macaques, which is a model of neuroHIV, causes a strong upregulation of TATA-box binding peptide (TBP) and components of the transcription initiation complex in brain microglia, suggesting that a mechanism of gene transcription that involves TATA box may be stimulated by the infection.

HIV-1 proviral transcription starts with the binding of cellular factors, such as NF-κB and Sp1, as well as the TATA box binding protein (TBP), which is a key component for recruiting RNA Pol II to the LTR. This complex allows the production of viral transcripts, which are then spliced and translated. Epigenetic and other limiting host factors, as well as the level of cellular activation, can potentially regulate such process. Tat binds to the trans-activator response (TAR) element hairpin at the 5′ end of viral RNA transcripts (16–19), and to the apical loop, to which the transcriptional elongation factor pTEFb binds in a Tat-dependent manner (20, 21). Upon TAR binding, the kinase component of pTEFb, cyclin-dependent kinase 9 (CDK9), can phosphorylate the C-terminal domain of RNA Pol II (21, 22). The pTEFb also directs the recruitment of TATA box binding protein (TBP) to the LTR, stimulating the assembly of new transcription complexes (23). Core promoter elements participate in the initiation factor TFIID complex, where TBP works as an anchor for TBP-associated factors (TAFs) 4–12 (24). The TFIID complex then makes contact with acidic activator proteins such as Sp1 and the initiator, while TBP recognizes the TATA box. Thus, TBP is a key element in the ability of Tat to recruit the host transcriptional machinery in favor of viral transcription.

The activation of the LTR-directed gene expression by Tat has been observed in CNS-derived cells, and in astrocytic glial cell lines (25). Importantly, it has been demonstrated that this can happen in the absence of a TAR element (25), suggesting that alternative mechanisms may exist. Importantly, HIV Tat can strongly upregulate inflammatory cytokines in the brain, *per se* mimicking aspects of the inflammatory outcomes observed in HIV infection (26, 27). In addition, it has been demonstrated that the ability of Tat to initiate transcription of heterologous genes through the TATA-box element can happen in the absence of any HIV-1 sequence, through mechanisms that are similar to DNA sequence-specific transcription factors (28, 29). This suggests that HIV-1 Tat may have the ability to enhance genes that present a TATA-box promoter element. While the HIV Tat peptide *per se* has the ability to stimulate a diversity of genes, both *in vivo* (15, 26, 27, 30), and *in vitro* (31–37), the contribution of the TATA-box promoter element to upregulated heterologous transcripts has not been examined.

The TATA box is the most well-studied core promoter element. The canonical TATA-box sequence, TATAAAA, may be variable in natural promoters (38, 39). Methodologies to estimate the frequency of TATA box-containing promoters also vary. As a result, there is a wide-range of estimates of how many genes have a Tata-box promoter motif [revised by (40)]. It is most accepted that the TATA-containing promoters make up only 10–16% of the genes read by RNA Pol II, of which only 30% contain the canonical TATA box (41). Systematic analysis of genes that bear this promoter element has pointed to specific biological processes, associated to organogenesis and morphogenesis, with an impact in early development, but also has found a second largest contribution to responses to stimulus and to stress, as well as to defense, supporting the role of TATA-box mediated transcription in the elevation of early-response, immune and inflammatory genes. TATA-less promoters, on the other hand, tend to be involved in basic biological maintenance processes, or be constitutively expressed (40, 42).

Here, we have examined the hypothesis that the HIV-1 Tat protein has the property of increasing inflammatory cytokines in innate immune cells through favoring a TATA box-supported mechanism, and that this mechanism may be enhanced by Meth exposure. Using the THP1 innate immune cell line system, we show evidence that the TATA box-binding transcription factor TBP is increased by HIV-1 Tat, along with the recruitment of RNA PolII to promoters of genes that bear the TATA-box promoter. We also show that this effect is enhanced when HIV-1 Tat is combined with Meth.

The lack of TAR in host gene promoters argues against a direct interaction between TBP and Tat. Yet, binding of HIV Tat to the host chromatin has been described in T cells (43), where it functions through master transcriptional regulators bound at promoters and enhancers, rather than through cellular “TAR-like” motifs, to both activate and repress gene sets sharing common functional annotations (44). The functional interaction between HIV-1 Tat and TBP has been proposed in different models (29, 45, 46). In our system, we have not tested whether Tat plays a role at bridging TBP and its binding motifs, or whether Tat just activates TBP as major transcription factor in the innate immune environment. However, we have found evidence that Tat is able to translocate to the nucleus soon after cell exposure.

We have developed a strategy to test the hypothesis of TBP as a major transcription factor in the activation of TATA-box-bearing inflammatory genes by HIV Tat *in vitro*, by engineering a THP1 cell line with impaired transcription of TBP dominant splice variants. As a result, we produced a human innate immune cell line with a deficient expression of TBP protein. In that cell line, we have found that TBP was critical for the upregulation of genes stimulated by HIV Tat, including inflammatory molecules. Yet, in the presence of Meth, alternative transcriptional factors positively enhanced a significant fraction of genes with TATA box motifs, bypassing the TBP disruption.

Our findings demonstrate that TBP is indeed a critical transcription factor in Tat-induced host responses that can result in pathogenesis, but co-morbidities such as the stimulant drug Meth, can increase the transcriptional complexity and promoter plasticity, downgrading the central role of TBP in inflammation.

## Materials and Methods

### THP1 Cell Culture

THP-1 cells were obtained from ATCC (TIB-202). They were maintained in RPMI 1640 containing 2 mM glutamine; 10 mM HEPES; 1 mM sodium pyruvate; 4.5 g/L glucose; 1.5 g/L sodium bicarbonate; 0.05 mM beta2 mercaptoethanol, as well as 10% heat inactivated fetal bovine serum (Hyclone), and 100 ug/ml Pen/Strep.

### *In vitro* Treatments With HIV-1 Tat and With Meth

For treatments, the THP1 cells were plated at 2 × 10^6^ cells/well in 12 well plates, containing 2 ml per well. The HIV-1 IIIB Tat Recombinant Protein (MW: 14,000) was obtained from the NIH AIDS Reagent Program. The doses used were 1–100 ng/ml for Western Blots, and 10 ng/ml for all the other assays. Meth was used at 60 μM, following previously optimized protocols, and for mimicking abuse concentrations in plasma (47). The cells were treated with either Vehicle, HIV-1 Tat, Meth, or HIV-1 Tat and Meth. The concentrations of Tat in culture were 1, 10, and 100 ng/ml. The concentrations of Meth were 30 μM, 60 μM (shown in Figures), and 120 μM, which produced similar results regarding the lack of TBP induction. The cells were then harvested 15 min after exposure for ChIP assays, and 2 h for Western blots, RNA-seq and validations. These time points were selected following a time-course that demonstrated that epigenetic events that result directly from Tat and/or Meth, and not from factors produced by the cells as a result from the stimulation, are detectable in 15 min. Yet, a 2 h period is necessary for confirming productive events at the transcriptional and protein level. All experiments were performed at least 3 times, in triplicate, otherwise stated in the figure legend.

### Western Blots

The cells treated as described were centrifuged at 2,500 g for 5 min, and the pellets were washed twice in PBS, and in 0.5% Triton X buffer, for isolation of nuclear fraction, before being resuspended in RIPA buffer (Thermofisher), added 1 mini tablet Complete (Roche protease inhibitors) for protein extraction. Western blots for detection of TBP and TAF9 were performed by loading 20 μg of whole cell lysates and 5 μl of PageRuler Plus Prestained Protein Ladder (Thermofisher) per well, onto a Mini-Protean TGX 4–20% pre-cast gel (Biorad). Proteins were transferred to a nitrocellulose membrane using a G2 Blotter, and blocked with 5% Casein in TBST for 1 h at room temperature. The N-terminus of the TATA binding protein (TBP) was detected at 36–38 kD using a TBP monoclonal antibody (1TBP18/ MA1-189) at a dilution of 1:1,000. TATA box associated factor 9 (TAF9) was detected at ~20 kD using a polyclonal antibody (PA5-40919), at 1:1,000. Both antibodies were incubated overnight at 4oC on a rocking platform, followed by a Goat anti-Mouse IgG (H+L) HRP conjugate (Cell Signaling) for 1 h at room temperature. Chemiluminescent detection was performed using SuperSignal Pico substrate (Thermofisher).

### TBP and Tat Nuclear Translocation

The THP1 cells were brought to the concentration of 10^6^/ml and stimulated with 50 nM of phorbol-12-myristate-13-acetate (PMA) for 24 h for differentiation, prior to the stimulation with Meth (30 or 60 μM), and/or HIV Tat (10 ng/ml). The cells were fixed with 4% paraformaldehyde, 5, 15, 30, and 60 min following stimulation, and then were washed with PBS. The wells were then treated with PBS containing 0.1% Triton X-100 for 20 min at room temperature, rinsed 3 times with PBS, and then blocked with 5 g/l Casein (Sigma Aldrich) in PBS, containing 0.5 g/l Thimerosal (Sigma Aldrich) for 1 h at room temperature. The primary antibody against TBP was a rabbit polyclonal (HPA049805, Sigma Aldrich), and the antibody anti-Tat peptide was obtained through the NIH AIDS Reagent Program, Division of AIDS, NIAID, NIH: Anti-HIV-1 Tat Monoclonal (15.1) from NIAID, DAIDS (cat# 1974). Both antibodies were diluted in blocking solution, and then placed overnight, at 4°C, with gentle rotation. Then cells were rinsed 3 times for 10 min with 1% blocking solution in PBS, containing 0.1% Triton-X, followed by incubation with a secondary AlexaFluor 647-labeled anti-rabbit or anti-mouse IgG (Thermo Fisher Scientific), for 2 h at room temperature, in the dark. After rinsing, 4′, 6-Diamidino-2-Phenylindole, Dihydrochloride (DAPI) was diluted to 300 ng/ml in 1% blocking solution for 2 min, in the dark. Cells were rinsed and maintained in PBS, and observed in a Nikon A1R laser-scanning confocal mounted onto a Nikon inverted Ti-E scope (Nikon, Melville, NY), and with a 20x PlanApo objective, 0.8NA (Nikon) and Images were acquired using a NIS-Elements C software (Nikon). Fluorescence intensity was normalized against background (secondary antibody only). Image analysis was performed in Fiji/ImageJ (National Institute of Health, USA). For that, tiff image files from separate channels were opened, transformed into 32-bit images, and manually thresholded to identify DAPI nuclei, and stained cells. A binary mask was obtained from the negative thresholded DAPI image and subtrated from the total transcription factor stained area. The translocation index was calculated as percentage of the total transcription factor stained measurement values that is co-localized within the nuclear area, and derived from the difference between total and nuclear staining. The colocalization was further confirmed in calculated images where 32-bit thresholded channels were averaged.

### Fixation for Chromatin Immunoprecipitation

The cells were fixed with a 1.1% Formaldehyde (Sigma Aldrich F-8775), and molecular grade 0.01 M NaCl, 0.1 mM EDTA pH 8, 50 mM HEPES pH 7.9, in H_2_O. The plate was then agitated on a titer plate shaker for exactly 15 min at room temperature. The fixation was then interrupted with 125 mM Glycine (Sigma Aldrich G-7403). The samples were let set at room temperature for 5 min. After the glycine incubation, the cells were transferred to conical 15 ml tubes and kept on ice for the remainder of the procedure. The tubes were centrifuge at 800 × g at 4°C for 10 min, the supernatants were removed and the cells were resuspended in 10 ml ice-cold PBS-Igepal (PBS containing 0.5% Igepal CA-630 (Sigma Aldrich I-8896) per tube, by pipetting up and down. The tubes were centrifuged again as before, and pellets were again resuspended in 10 ml PBS-Igepal. Then, 100 μl from a PMSF stock (100 mM in ethanol) was added to each tube for a final concentration of 1 mM. The tubes were centrifuged a third time to pellet the cells, and the supernatant was completely removed from cell pellets. The pellets were snap-frozen on dry ice and store at −80°C, until further procedures. The pellets were sonicated and the DNA sheared to an average length of 300–500 bp. Genomic DNA (input) was prepared by treating aliquots of chromatin with RNase, proteinase K, and heat (65°C) to reverse cross-linking, followed by phenol and chloroform extractions and ethanol precipitation. Pellets were resuspended in 10 mM Tris, 1 mM EDTA, and the resulting DNA was quantified on a Nanodrop spectrophotometer (Thermo Fisher Scientific, Chicago, IL). Results were used to calculate the total chromatin yield.

### Chromatin Immunoprecipitation (ChIP) of RNA Pol II

RNA Pol II ChIP reactions were performed using 30 μg of human THP1 cell chromatin and 4 μg of antibody anti-RNA Pol II (Abcam, ab5095). Positive control primers (b-Actin and GAPDH) as well as negative control primer pairs were used to amplify regions in a gene desert on chromosome 12 and 4 (Untr12, Untr4) by qRT-PCR. Signals for RNA Pol II were strong, with enrichments of the best positive control signal over background up to 99-fold. For the library generation and sequencing, 50-nt sequence reads identified using Illumina's Hi-Seq, were mapped to the genome using the BWA algorithm with default settings. Alignment information to Human Hg19 for each read was stored in BAM format. Only reads that passed the purity filter, aligned with no more than 2 mismatches, and mapped uniquely to the genome were used in the subsequent analysis. Duplicate reads were removed. Since the 5′-ends of the aligned reads (= “tags”) represent the end of ChIP/IP-fragments, the tags were extended *in silico* (using Active Motif software) at their 3′- ends to a length of 150–250 bp. To estimate the density, the genome was divided into 32-nt bins and the number of fragments in each bin was determined, to provide a “signal map”; histograms of fragment densities were stored in both a BAR file, for viewing in Integrated Genome Browser (IGB), and bigWig file, uploaded to the UCSC Genome Browser. BAR/bigWig files also provide the peak metrics in the Active Motif analysis program as described below. Intervals, describing genomic regions with local enrichments in tag numbers, were defined by the chromosome number, a start and end coordinates. SICER (48) was used to study RNA polymerase II binding to extended regions in the genome, by looking for significant enrichments in the ChIP/IP data file when compared to the Input data file (random background), and also for examining transcription factor binding sites using TRANSFAC Match weight matrix (49, 50). Peaks and false positives were estimated by thresholding of the BAR files. In the default analysis, the tag number of all samples is reduced (by random sampling) to the number of tags present in the smallest sample. Only Input/IgG control peaks that overlapped with Intervals in the ChIP/IP data were used in the analysis. After defining the Intervals and Active Regions, their genomic locations, their proximities to gene annotations, and other genomic features, were determined.

### ChIP Analysis

Genes with RNA Pol II peaks found within 10 kb of start/end were compared in the ChIP samples. Overlap between the samples was high (70–90% in pairwise comparisons), reflecting the fact that the majority of gene transcription does not change. There were 4,865 active genomic regions that presented overlapping intervals from all conditions. Active regions were identified for the comparison of all samples, and the peak metrics was used in combination with the present/absent peak call information to find genomic regions with RNA Pol II occupancy patterns of interest. Ratios of treated samples compared to their respective controls were used to average signal values (>1.5-fold change) and to perform transcription factor and pathway analysis using TRANSFAC, iRegulon and Genemania in Cytoscape, and iPathwayGuide. Regions of Pol II enrichment in ChIP-Seq data often did not correlate perfectly with the gene annotations. Typically, a stronger peak was seen at the transcription start site (TSS/promoter), and high enrichments were often seen extending into downstream regions. Therefore, the average fragment densities for 25,143 human genes (from TSS to termination site) and the corresponding promoters (from −1,000 to +1,000 nt relative to TSS) were determined. The values between different gene promoters were comparable because they were based on a 2,000 bp segment. Peak values that were found in gene bodies were corrected by length. Table 1 shows the total number of reads sequenced and their alignment to the human genome (hg19). For the analysis, the data files were normalized to the same number of unique alignments without duplicate reads, which was 16 million. Intervals were determined using the SICER algorithm at a cutoff of FDR1E-10 and a Gap parameter of 600 bp (which merges peaks located within 600 bp of each other). Gene intervals (peaks) were determined using the SICER algorithm at a cutoff of FDR1E-10 and a Gap parameter of 600 bp (which merges peaks located within 600 bp of each other into a single “island”). The number of intervals identified ranged from 9,775 in Meth-treated cells to 14,124 in cells treated with both Meth and Tat, within 10 kb of start/end. The overlap between the samples was high (70–90% in pairwise comparisons), reflecting the fact that the majority of gene transcription does not change. At 4,865 genomic regions, all conditions showed overlapping intervals. Because RNA Pol II- enriched regions are not typical bell- shaped, symmetrical peaks, average signal values were used in the analysis, and for estimating the ratios between each treatment condition and controls, and in additional pairwise comparisons. Regions of RNA Pol II enrichment in ChIP-Seq data did not correlate perfectly with the gene annotations, but as expected, stronger peaks were typically seen at the transcription start site (TSS/promoter), and high enrichments are often seen extending into the downstream region. Thus, the tag densities/signal metrics relative to the known gene annotation were examined. In this analysis, the average fragment densities for 25,143 human genes (from TSS to termination site) and the corresponding 25,143 human promoters (from −1,000 to +1,000, relative to TSS) were determined. The values between different gene promoters were comparable since they were based on a 2,000 bp segment.

**Table 1:** Number of sequenced reads and their alignment to the human genome (hg19).

	**Control**	**Meth**	**Tat**	**Meth + Tat**	**Pooled Input**
Total number of reads	35,582,719	36,449,122	23,742,921	40,019,315	35,838,803
Total number of alignments	32,594,991	34,255,959	21,211,764	36,906,854	34,240,934
Unique alignments (-q 25)	27,536,469	28,943,392	17,774,903	31,197,538	28,385,207
Unique alignments (without duplicate reads)	22,337,553	24,631,143	15,956,453	27,319,946	25,642,285
Normalized	15,956,453	15,956,453	15,956,453	15,956,453	15,956,453

### Production of TBP-Knock Out THP1 Clone by CRISPR/Cas9

A template for disruption of the TBP gene (ENST00000412512, NM001172085) was established following described methods (51–54), and based on the conserved protein coding sequence in 8 detected splice variants (ENST00000423353.1, 703 bp). Primers for detection of human TBP and GAPDH by SyBrGreen qRT-PCR were purchased from Qiagen (Germantown, MD). A guide sequence was designed in http://crispr.mit.edu/guides, by introducing the FASTA sequence of the TBP coding region onto the target human genome (h19). Twenty-six high score guide sequences targeting exon 1 coding region mapped to the correct transcript, from which one was selected for oligo synthesis (sgTBP#1.1 AGCCTGCCACCTTACGCTCA), by Integrated DNA Technologies (San Diego, CA). For nickase Cas9, gRNA left GATAGGGATTCCGGGAGTCA, and gRNA right CCAATGATGCCTTATGGCAC were selected. Using the protein coding sequence data from ENSEMBLE and the designed gRNAs from the CRISPR database, the guide binding pattern was mapped out, identifying 5 out of 7 total wild-type gRNA targets transcripts, and 6 out of 7 nickase gRNA targets, with 0 off targets. The gRNAs and the Cas 9 protein were put into a plasmid for expression, using a GFP tag for assessing efficiency and selecting positively transfected cells by flow cytometry. For that, we used the CRISPR/Cas9 gesicle technology (55), with Guide-It CRISPR/Cas9 gesicle system (Clontech, Mountain View, CA). First, the oligos were annealed and cloned into pGuide-it-sgRNA1 vector, following manufacturer's protocols. Positive colonies were picked and expanded in LB, and plasmid DNA was isoladed using NucleoBond Xtra Midi (Clontech), and sequencing analysis was performed by Eton Bioscience Inc (San Diego, CA), using provided Guide-it sequencing primer 1. The gesicle-producing 293T cell line (Clontech) was expanded in DMEM with high glucose (4.5 g/l) (Genesee, San Diego, CA), 4 mM L-glutamine, and sodium bicarbonate, 10% fetal bovine serum Hyclone (GE Healthcare Lifesciences, Pittsburgh, PA), and 1% Penicilin/Streptomycin (Genesee), and split using Trypsine-EDTA (Sigma-Aldrich). These cells were seeded in 10-cm tissue culture dishes treated with poly-L-Lysine (Corning). Then 10 μg of plasmids encoding for sgRNA mixed to a plasmid containing Cherry picker red fluorescence, and the Cas9 enzyme were applied to the 293T cells, following manufacture's protocols. The A/C heterodimerizer ligand was added to the cultures for driving active loading of the Cas9/sgRNA complex into gesicles. A similar procedure, using Cas9 without the sgRNA was performed for producing “empty” gesicles, used for control conditions. Seventy-two hours after that, transfected cells were visualized due to Cherry picker red fluorescence. The cell media was harvested, and centrifuged overnight at 8,000 × g in a Beckman J2-HS centrifuge, with a JS 7.5 swinging rotor, and the pellet was concentrated to ~100 μl final volume in PBS. The gesicles were placed at 4°C on a rocking platform for 2 h, and stored in aliquots at −80°C until ready for treating THP1 cells. The cells were resuspended in protamine medium (containing 8 μg/ml protamine sulfate, Sigma), plated at 2 × 10^5^ cells/ well in 500 μl, in 24 well plates, and 30 μl of gesicles were added. The plates were centrifuged at 1,150 × g for 30 min at room temperature. The plates were incubated at 37°C overnight, and then positively transfected cells were visualized under a fluorescence-microscope for detection of intracellular red fluorescence signal originated from the Cherry Picker reporter on the gesicle surface. The Cherry Picker-positive cells from each positive well were washed in HBSS without phenol red, containing 10% fetal bovine serum, 1% penicillin/streptomycin, and resuspended in this buffer for sorting using a BD FACSJazz (BD Biosciences, San Diego, CA). Sorted positive cells were expanded into clones (1A, 1B, 2A, 2B, 2C, and 8), for at least 7 days before testing the TBP mutation. The detection of the TBP mutation was tested using DNA hybridization with Guide-It Resolvase (Clontech), on the gel-purified TBP band, amplified from QIAmp-extracted genomic DNA (Qiagen), and following manufacturers' protocols. The TBP gene was amplified with primers 5′ GAGTTCCAGCGCAAGGGTT forward, and 5′ TTTTGCAGCTGCGGTACAAT, using Phusion High Fidelity DNA Polymerase (New England Biolabs, Ipswich, MA), following manufacture's protocol, to generate two fragments, one 63 bp and one 465 bp, visualized on 2% agarose gel. Clone 2A presented efficient TBP mutagenesis and was used for the comparison with control clone C, which was generated as above, but with empty gesicles. Clone 1A had poor mutagenesis, and was used in some experiments as a control in addition to clone C. The TBP gene disruption was further tested at the mRNA level, which was extracted from cell pellets using RNeasy Mini kit (Qiagen), and reverse-transcribed with Superscript II (Thermo Fisher). The presence or absence of the TBP transcript confirmed the efficacy of CRISPR/Cas9 to disrupt the TBP gene (Figure 4).

### Treatment of TBP-Mutant Clone 2A and TBP-Sufficient Clone C

The TBP-deficient clone 2A and control clone C were expanded in RMPI 1640, containing 10% fetal bovine serum (Hyclone), 1% penicillin/streptomycin, and 25 mM HEPES (Invitrogen). The cells were plated in 24-well plates at 2 × 10^6^ cell/ml, and then stimulated with 10 ng/ml of HIV-1 IIIB Tat Recombinant Protein (NIH AIDS Reagent Program), 30 or 60 μM of Meth, or both HIV-1 Tat and Meth together. The cells were then harvested 2 h after stimulation, and RNA-seq was performed.

### RNA-Seq Protocol

RNA was isolated from TBP-deficient THP1 clone 2A, and TBP-sufficient control clone C using the RNeasy Mini Kit (Qiagen) with on-column DNAse I treatment. quantified using Qubit 2.0 Fluorometer (Life Technologies, Carlsbad, CA, USA). The eluted RNA had a 260/280 of greater than 1.8. RNA integrity was checked with Agilent TapeStation (Agilent Technologies, Palo Alto, CA, USA). RNA samples were quantified using Qubit 2.0 Fluorometer (Life Technologies, Carlsbad, CA, USA) and RNA integrity was checked with Agilent TapeStation (Agilent Technologies, Palo Alto, CA, USA). RNA library preparation, sequencing reaction, and bioinformatics analysis were conducted at GENEWIZ, LLC. (South Plainfield, NJ, USA). RNA sequencing library preparations used the NEBNext Ultra RNA Library Prep Kit for Illumina by following manufacturer's recommendations (NEB, Ipswich, MA, USA). Briefly, mRNA was first enriched with Oligod(T) beads. Enriched mRNAs were fragmented for 15 min at 94°C. First strand and second strand cDNA were subsequently synthesized. cDNA fragments were end repaired and adenylated at 3′ends, and universal adapter was ligated to cDNA fragments, followed by index addition and library enrichment with limited cycle PCR. The sequencing libraries were validated on the Agilent TapeStation (Agilent Technologies, Palo Alto, CA, USA), and quantified by using Qubit 2.0 Fluorometer (Invitrogen, Carlsbad, CA) as well as by quantitative PCR (Applied Biosystems, Carlsbad, CA, USA). The sequencing libraries were clustered on one lane of a flowcell. After clustering, the flow-cell was loaded on the Illumina HiSeq instrument according to manufacturer's instructions. The samples were sequenced using a 2 × 150 Paired End (PE) configuration. Image analysis and base calling were conducted by the HiSeq Control Software (HCS). Raw sequence data (.bcl files) generated from Illumina HiSeq was converted into fastq files and de-multiplexed using Illumina's bcl2fastq 2.17 software. One mis-match was allowed for index sequence identification. After investigating the quality of the raw data, sequence reads were trimmed to remove possible adapter sequences and nucleotides with poor quality using Trim Sequences Module in CLC Genomics Workbench 9.0.1. The trimmed reads were mapped to the Homo sapiens GRCh38 reference genome available on ENSEMBL using the RNA-Seq Analysis Module in CLC Genomics Workbench 9.0.1. BAM files, Unique gene hit counts and Unique transcript hit counts were generated as a result of this step. After extraction of gene and transcript hit counts, the hit counts tables were used for downstream differential expression analysis. Using Kal's test, a comparison of gene expression between the groups of samples was performed. The Kal's test was used to generate *p*-values and Log2 fold changes. Genes and transcripts with adjusted *p*-values < 0.05 and absolute log2 fold changes > 1 were called as differentially expressed genes and transcripts, respectively, for each comparison. A gene ontology analysis was performed on the statistically significant set of genes by implementing the Hyoergeometric tests in CLC Genomics Workbench 9.0.1. The goa human GO list was used to cluster the set of genes based on their biological process and determine their statistical significance. Sequencing performance was assessed for total number of aligned reads, total number of uniquely aligned reads, and genes detected. All gene counts were then imported into the R/Bioconductor package EdgeR and TMM normalization size factors were calculated to adjust for samples for differences in library size, and then imported into R/Bioconductor package Limma. Performance of the samples was assessed with a Spearman correlation matrix. Weighted likelihoods based on the observed mean-variance relationship of every gene and sample were calculated with the voom WithQualityWeights function and gene performance was assessed with plots of residual standard deviation of every gene to their average log-count with a robustly fitted trend line of the residuals. Generalized linear models were created to test for gene level differential expression, and the results were displayed with FDR *p*-values ≤ 0.05 and log 2-fold-changes greater than an absolute value of 2, in each given comparison.

### Expression Data Analysis

Using the Cytoscape interface platform, Mentha and Biogrid were used for building a network, using transformed fold change and FDR corrected *p*-values as attributes to visualize the effects of TBP deficiency in changes caused by 2 h of stimulation with HIV Tat, in the presence or absence of Meth. BinGO was used to estimate pathways affected in cells that were engineered to lack TBP expression, following stimulation.

## Results

### TBP Is Activated and Translocated by HIV-1 Tat in Human THP1 Macrophages

**Figure 1:**
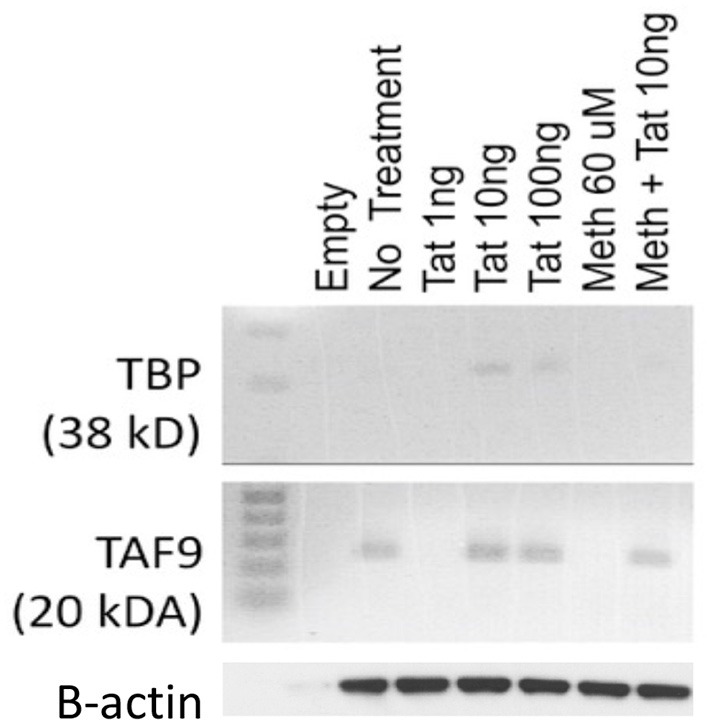
Levels of TBP and TAF9 in the nuclear fraction of THP1 cells stimulated with HIV-1 Tat and with Meth. THP1 cells were incubated with different concentrations of HIV-1 Tat peptide (1, 10, and 100 ng/ml), and with Meth (60 μM). Protein was extracted from nuclear pellets 2 h after stimulation.

In order to test the hypothesis that the HIV-1 Tat may utilize a TATA-box mediated mechanism, which may be involved in the ability of that protein to activate transcription of inflammatory genes that have a TATA-box promoter domain, we used a human macrophage cell line, THP1. These cells can activate the TBP protein when treated with 10 and 100 ng of the HIV-1 Tat peptide for 2 h, but not with 1 ng (Figure 1). Meth alone did not activate TBP at any concentration, but when combined with Tat, there was a detectable increase (Figure 1). The optimal concentration of Tat for activation of TBP was 10 ng/ml, which was the concentration maintained throughout the experiments. Other components of the TFIID complex, such as TAF9, were increased by Tat, with or without Meth, but not by Meth alone (Figure 1).

**Figure 2:**
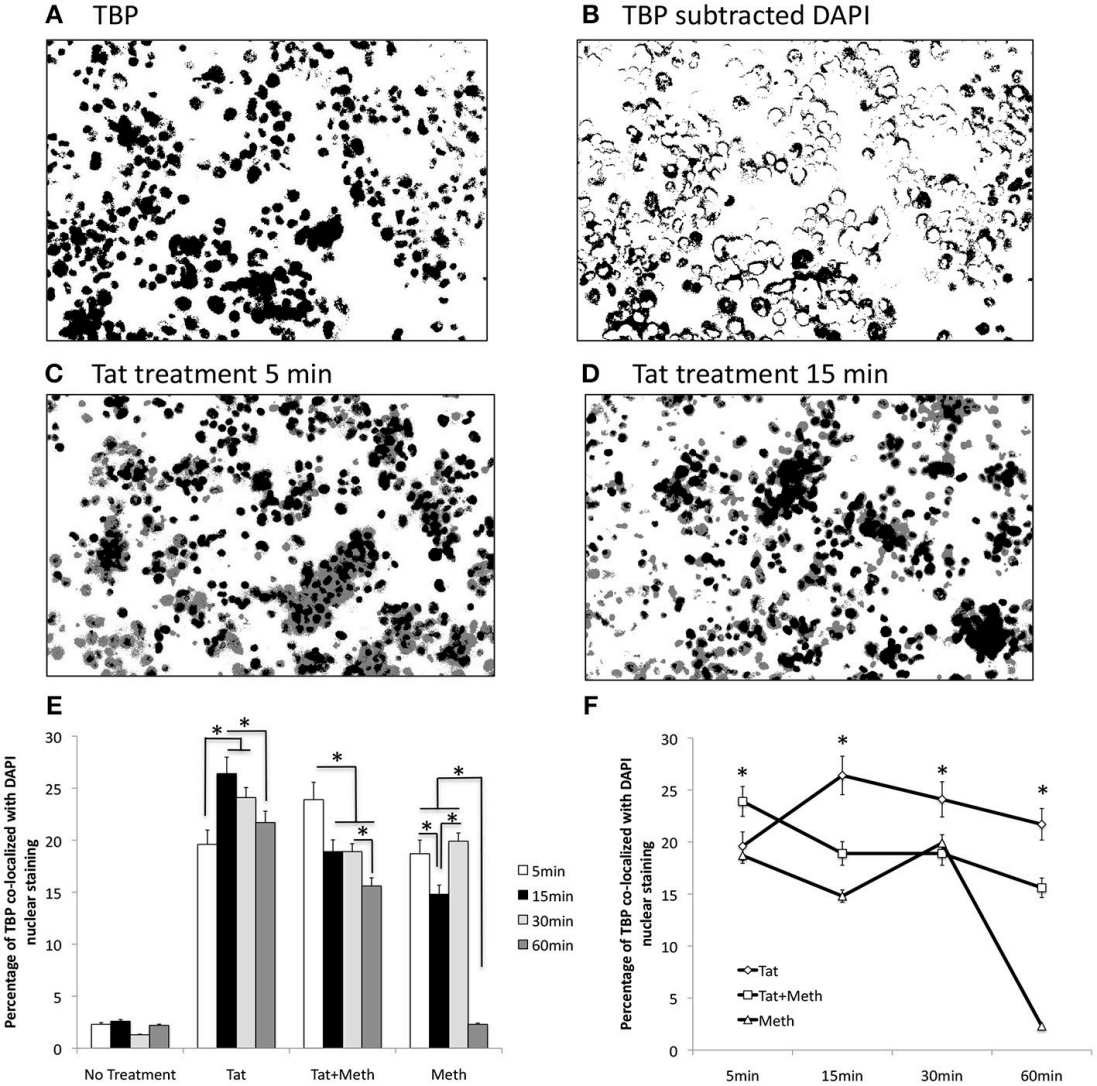
TBP translocation patterns by Tat and by Meth. Translocation was estimated by immunocytochemistry on differentiated THP1 cells that were treated with HIV-1 Tat (10 ng/ml), and/or Meth (60 μM), over 5, 15, 30, and 60 min. Acquired images of cells stained with anti-TBP (rabbit polyclonal), and anti-rabbit AlexaFluor 647, and counterstained with DAPI are in Supplementary Materials. The images were digitalized as described in Methods. DAPI staining was converted into a binary mask and subtracted from the total TBP staining **(A)**, to produce an image of cytoplasmatic staining **(B)**. The average of overlapped binary masks confirmed the co-localization of TBP and DAPI upon Tat stimulation at 5 min **(C)**, and with a peak at 15 min **(D)**. After measurements, the translocation index was calculated as the percentage of the total staining that was co-localized with DAPI. **(E)** Bar graph showing translocation indexes for baseline untreated cells, and cells treated with all stimulants over time. **(F)** Line graph for visualization of the effect of time on TBP translocation with the different stimuli. The assays were performed in triplicate, and in three independent experiments. Results represent the average and standard deviation of 3 experiments performed in duplicate. *p < 0.05 in two-way ANOVA, followed by Bonferroni's test.

We tested the ability of Tat and Meth to translocate TBP by immunocytochemistry, using image analysis strategies illustrated in Figures 2A,B, and described in Methods. Representative original images are in of Supplementary Figure 1. The treatment of THP1 cells with Tat was able to translocate TBP into the nucleus, detectable since 5 min following the exposure (Figure 2C), and maintained TBP translocation throughout the analysis period, with a peak at 15 min (Figure 2D). On the other hand, Tat together with Meth induced a stronger translocation at 5 min, but that was gradually decreased. Meth alone also induced TBP translocation at 5 min, but this was quickly decreased and returned to baseline levels at 60 min (Figures 2E,F).

**Figure 3:**
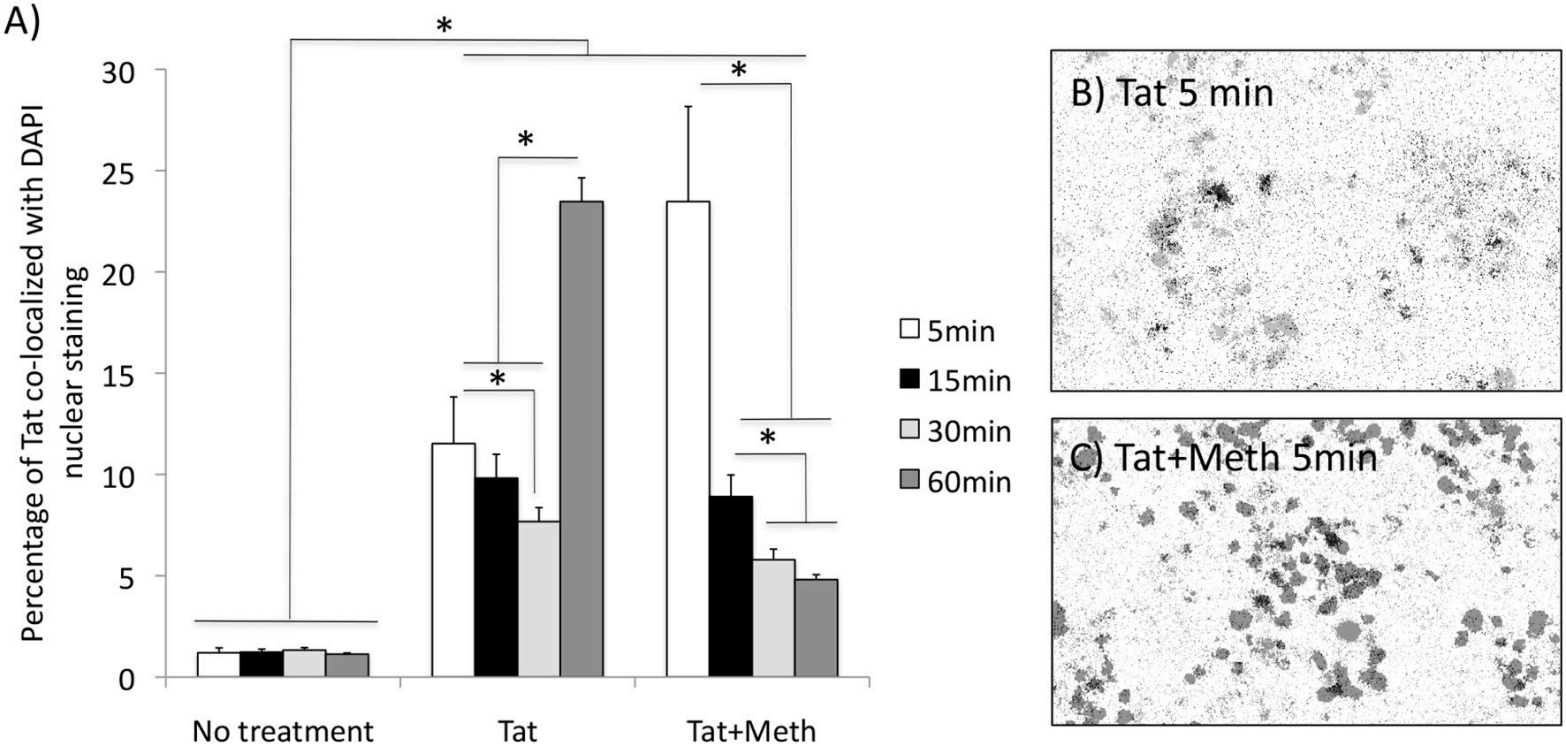
Tat translocation patterns in the presence of Meth. Translocation was estimated by immunocytochemistry on differentiated THP1 cells that were treated with HIV-1 Tat (10 ng/ml), or Tat together with Meth (60 μM), over 5, 15, 30, and 60 min. The cells were stained with anti-Tat (Monoclonal 15.1), and anti-mouse AlexaFluor 647, and counterstained with DAPI. The images were digitalized as described in Methods. DAPI staining was converted into a binary mask and subtracted from the total Tat staining. **(A)** Measurements were used for calculating the percentage of the total Tat staining that was co-localized to DAPI. Results represent the average standard deviation of 3 experiments performed in duplicate. **(B)** Representative image of the averaged overlapped binary masks, which confirmed the co-localization of Tat and DAPI upon Tat stimulation at 5 min), and **(C)** enhancement by Meth simultaneous stimulation. *p < 0.05 2-way ANOVA, followed by Bonferroni's *post hoc* test.

We have also tested whether Tat is able to translocate to the nucleus. Figure 3A shows that Tat enters the cellular nucleus soon after exposure and over time. The acute translocation of Tat was enhanced by Meth at 5 min, but this was followed by a gradual extinction effect. Figures 3B,C show the channel average overlap confirming the entry of Tat (black) into the nuclear structure (gray).

### RNA Pol II Recruitment to Promoters Containing the TATA-Box Element Is Increased by HIV-1 Tat, and Further Enhanced by Meth

In order to examine the effects of Tat, and of the interactions with Meth on early transcriptional modifications in innate immune cells, we have used ChIP-seq to estimate changes in RNA Pol II recruitment to gene promoter regions, and to determine commonalities in transcription factor regulation. For that, THP1 macrophages were stimulated with 10 ng/ml of HIV Tat and/or 60 μM of Meth. The cell pellets were examined 15 min after stimulation, for identification of changes that are immediately due, and specific to, the provided stimuli, rather than due to secondary autocrine cytokine stimulation.

The examination of active promoter regions revealed that the majority of the differences in RNA Pol II signal enrichment between conditions were not above 2-fold, except for cells treated with Meth, which showed strongest signals (Supplementary Materials—Active Regions).

#### Visualization of Changes in RNA Pol II Recruitment Patterns

Using Cytoscape interface platform, Mentha and Biogrid interactomes were imported from the Proteomics Standard Initiative Common QUery InterfaCe (PSICQUIC) (www.bioconductor.org). These two databases were used for building a network from RNA Pol II recruitment data, ratio between experimental and controls' peaks signal average, and FDR-corrected *p*-values as node attributes, for visualizing changes.

**Figure 4:**
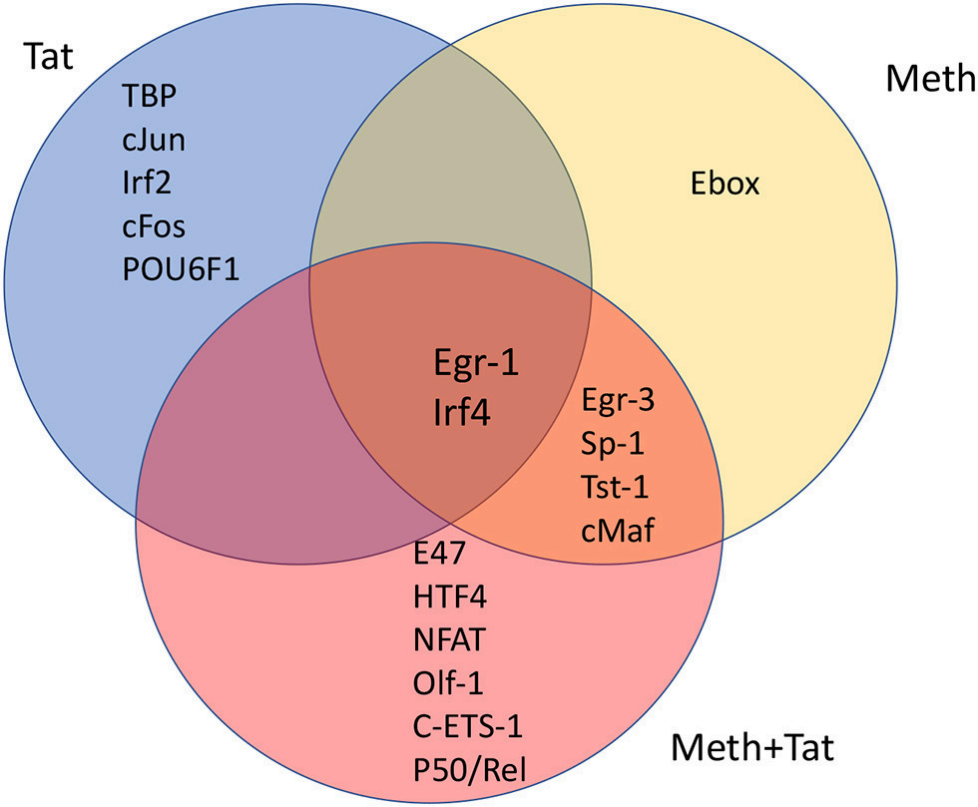
Venn's diagram showing transcription factors with frequent binding motifs among genes with significant changes in RNA Pol II recruitment, immediately following the stimulation of THP1 cells with HIV-1 Tat, Meth, or both together. Matrices for transcription factor binding motifs were generated from ChIP-seq data. For that, SICER raw data in BED format was loaded into TRANSFAC, and the Match-algorithm, was applied as binding factor identifier. Transcription factors with highest frequency of binding motifs in gene promoters affected by HIV-1 Tat, Meth, or both together were estimated. The transcription factors listed in each segment of the Venn's diagram are all significantly increased in comparison to control conditions, and are ranked in order according to frequency matrices, from highest on top to lowest at the bottom of the segregated lists.

The network was filtered for eliminating genes that were not represented in our data set. Gene networks were 63.73% due to co-expression, followed by 15.84% due to physical interactions. The organic layouts were restricted to selected default pathway database attributes (combined Wu-Stein-2010, PathwayCommons NCI Nature, IMID, Reactome and Cell_Map), which connected 5.27% of the genes (Figure 4). The application of attributes and visualization in Cytoscape suggested that Meth had a stronger effect on RNA Pol II recruitment when compared to HIV-1 Tat (Figure 4). Pathway perturbation and over-representation analysis by iPathwayGuide, showed that RNA Pol II recruitment was disturbed by Tat, mainly on genes involved in metabolism, activation and response to stimulus, such as cell adhesion molecules (*p* = 0.023), fatty acid elongation (*p* = 0.024), sulfur-relay system (*p* = 0.026), hematopoietic lineage (*p* = 0.034), Notch-signaling pathway (*p* = 0.04), purine metabolism (*p* = 0.043), systemic lupus erythematous (*p* = 0.046), and tryptophan metabolism (*p* = 0.049). Meth promoted RNA Pol recruitment to genes involved in metabolic pathways, such as fatty acid elongation (*p* = 0.007), glycan degradation (*p* = 0.007), DNA replication (*p* = 0.022), endocrine and other factor-regulated calcium reabsorption (*p* = 0.028), ribosome biogenesis (*p* = 0.039), NOD-like receptor signaling pathway (*p* = 0.048) and synaptic vesicle (*p* = 0.048).

We investigated whether promoters where RNA Pol II recruitment was changed by HIV-1 Tat, or Meth, or by both factors together, displayed critical promoter sequence commonalities, based on alignment and frequency of transcription factor binding motifs. This was examined using a combination of strategies, aiming at identifying common transcription master regulators potentially orchestrating the observed effects. First, SICER BED files were analyzed using MATCH tool algorithm (50) with h19 genome as a template, and using a library of mononucleotide weight matrices from TRANSFAC database (Wolfenbuttel, Germany). This tool created nucleotide matrices that calculated frequency of promoter binding motifs among genes exhibiting significant changes in RNA Pol II recruitment. Figure 4 shows the identified condition-specific transcription factors, in order of importance, in a Venn diagram. The attributed matrix score identified TBP (V$TATA_01, V$TATA_C, V$TBP_Q6, V$TBP_01, V$TBP_06), as the main transcription factor regulating changes upon HIV-1 Tat exposure, as determined by a consensus sequence matrix (Table 2). TBP was followed by other exclusive factors, cJun, Irf2, cFos and POU6F1, which were also statistically significant, but occurred at lower frequency when compared to TBP. Meth stimulation drastically shifted RNA Pol recruitment patterns to genes with high frequency of Egr-3, Sp-1, Tst-1, cMaf, and Ebox binding motifs. Of these, the Ebox (CACGTG/CAGCTG) binding motif aligned exclusively in Meth alone, while the other factors were common to Meth+Tat stimulation. E47, HTF4, NFAT, Olf-1, cETS-1, and p50/Rel were the transcription factors exclusively associated to Meth+Tat stimulation.

**Table 2:** TATA box promoter element matrices obtained from THP1 cells stimulated with Tat or with Meth+Tat.

**Reverse Consensus**	**A**	**C**	**G**	**T**	**Complement Consensus**	**A**	**C**	**G**	**T**
**(A) Tat stimulation**									
T	4	9	2	29	N	15	10	15	4
T	3	11	1	29	G	8	6	27	3
T	11	2	1	30	N	8	7	21	8
T	2	4	0	38	N	6	13	18	7
A	25	8	0	11	T	5	4	11	24
T	1	5	0	38	A	38	0	5	1
A	24	11	4	5	T	11	0	8	25
N	7	18	13	6	A	38	0	4	2
N	8	21	7	8	A	30	1	2	11
C	3	27	6	8	A	29	1	11	3
N	4	15	10	15	A	29	2	9	4
**(B) Meth+Tat stimulation**									
T	1	1	1	20	N	5	5	8	2
T	0	0	0	20	T	3	2	3	12
T	0	0	0	20	A	18	0	0	2
A	15	2	1	2	T	2	1	2	15
T	2	0	0	18	A	20	0	0	0
A	12	3	2	3	A	20	0	0	0
N	2	8	5	5	A	17	1	1	1

*The frequency of nucleotides matching the TATA box promoter element was calculated on complementary and reverse genomic sequences, using TRANSFAC*.

The finding that TBP binding motifs were the most frequent in genes affected by Tat alone is in agreement with the initial finding of increase of TBP at the protein level. It suggests a key role for the TATA-box promoter element in innate immune responses to HIV. Table 2 shows the frequency of nucleotides comprising the TATA box element matrix compiled from individual genomic sites upon HIV-1 Tat stimulation, in the absence or presence of Meth. The matrices estimating the frequency of nucleotides at the correct positions, suggest that upon Tat stimulation 31.5% of the genes showing enriched RNA Pol recruitment have the TATA box promoter element sequence. In contrast, in Meth+Tat stimulated cells, the average was decreased to 12%. In those cells stimulated with both Meth and Tat, the predominant transcription factor sequence domain (30.6%) identified by TRANSFAC was Egr-1 (GCGCATGCG), followed by Egr-3 (GTGGGT/C), and Sp-1 (GGGGCGGGG), all classified as C2H2 zink finger proteins.

Interestingly, many genes affected by Tat alone and by Meth+Tat were the same. Yet, estimated master regulators differed in those conditions, with Egr-1 and Irf4 in common. These two factors were also identified in genes affected by Meth alone, in addition to Egr-3, Sp-1, Tst-1, and cMaf, which were shared with Meth+Tat. On the other hand, Tat alone and Meth+Tat had no common regulators (Figure 4).

**Figure 5:**
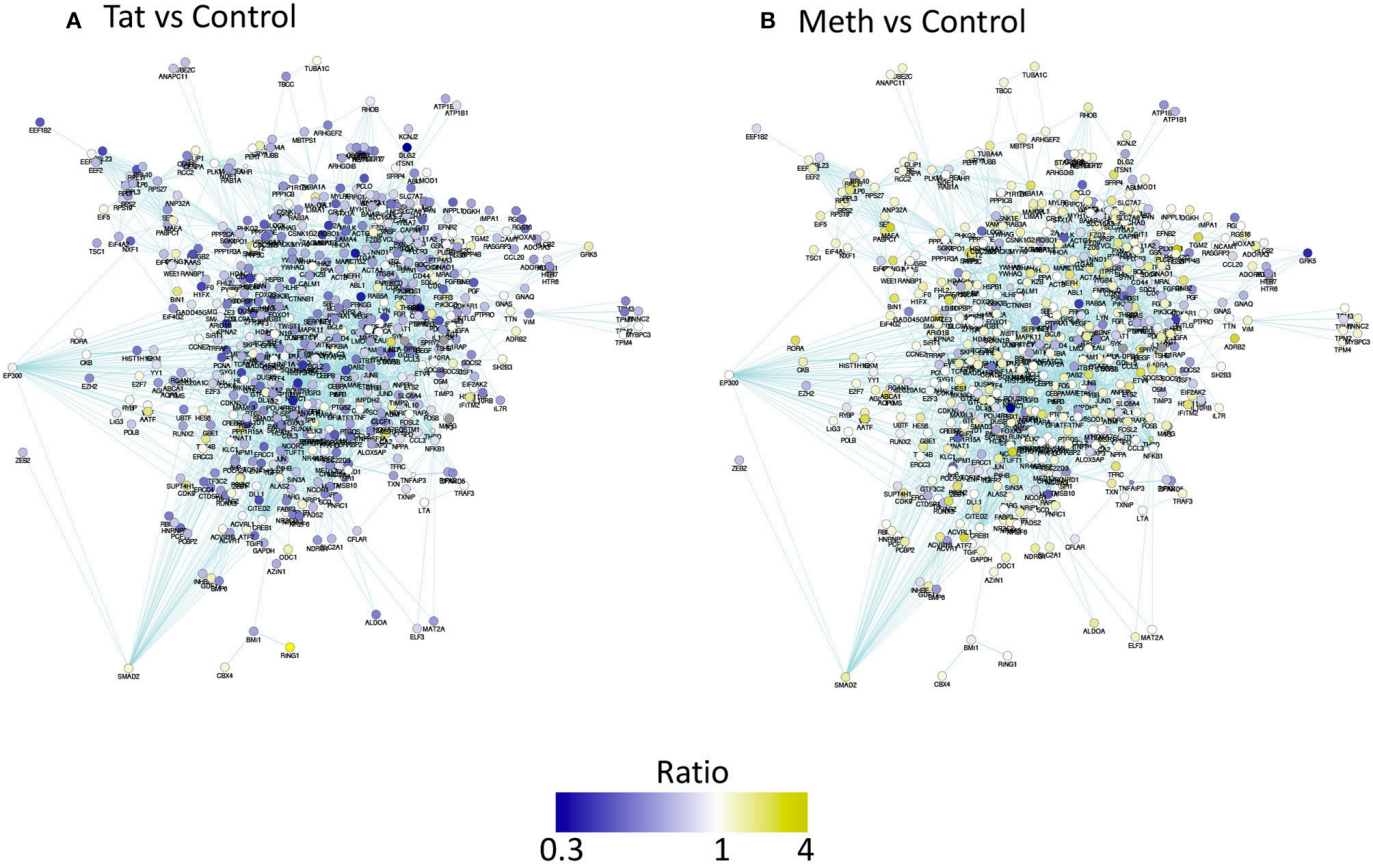
Pathway-based network association of genes with promoter activity changes due to HIV-1 Tat or Meth, compared to vehicle treatment. Ratio between RNA Pol normalized peak values between treated conditions and controls were used for designing a color-coded visualization. Genes were filtered to display only significantly decreased (blue), and increased (yellow) RNA Pol II recruitment patterns. **(A)** Visual representation of ratio of Tat over Control and **(B)** Meth over Control.

**Figure 6:**
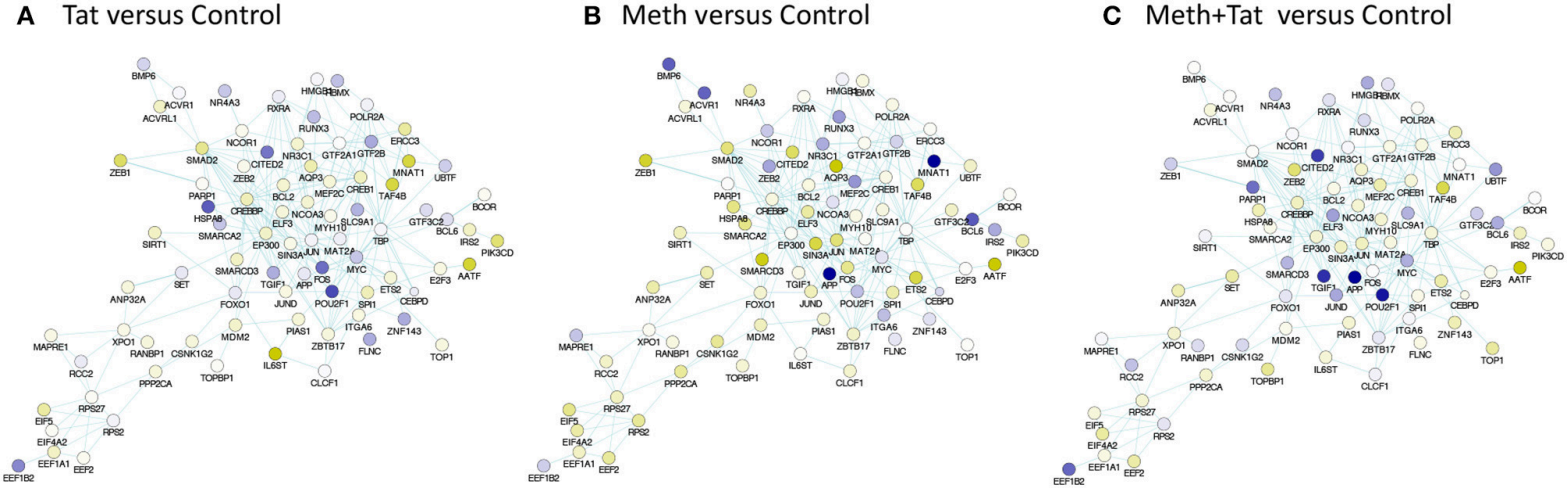
TBP-centered genomic neighborhood derived from pathway-based network association of genes with RNA Pol recruitment patterns affected by HIV-1 Tat or Meth. A sub-network was generated from the pathway-based association study through targeting and selecting the TBP gene and its first neighbors, through physical interaction edges. Colors designate significantly decreased (blue), and increased (yellow) RNA Pol II recruitment patterns, based on the ratio between **(A)** Tat over Control, **(B)** Meth over Control, and **(C)** Meth+Tat over Control.

The prediction of TBP as a master regulator of changes caused by Tat stimulation was further examined in iRegulon within the Cytoscape platform, using a targeted approach. This method identified all the genes displaying a TATA box element within ± 500 bp from the transcription starting site within the dataset. A table with predicted TBP-controlled genes and attributes was generated, and then utilized to produce a network in Genemania, for comparative analysis and visualization of critical differences between Tat and Meth stimulation (Figure 5). Overall, in Tat –stimulated THP1 cells there was a significantly higher number of genes with decreased RNA Pol II recruitment, while in Meth-stimulated cells, more genes were increased. However, upon the generation of a sub-network through targeting TBP and its first neighbors linked through physical interactions, most genes associated with that transcription factor had increased recruitment of RNA Pol II with all treatments (Figure 6).

**Figure 7:**
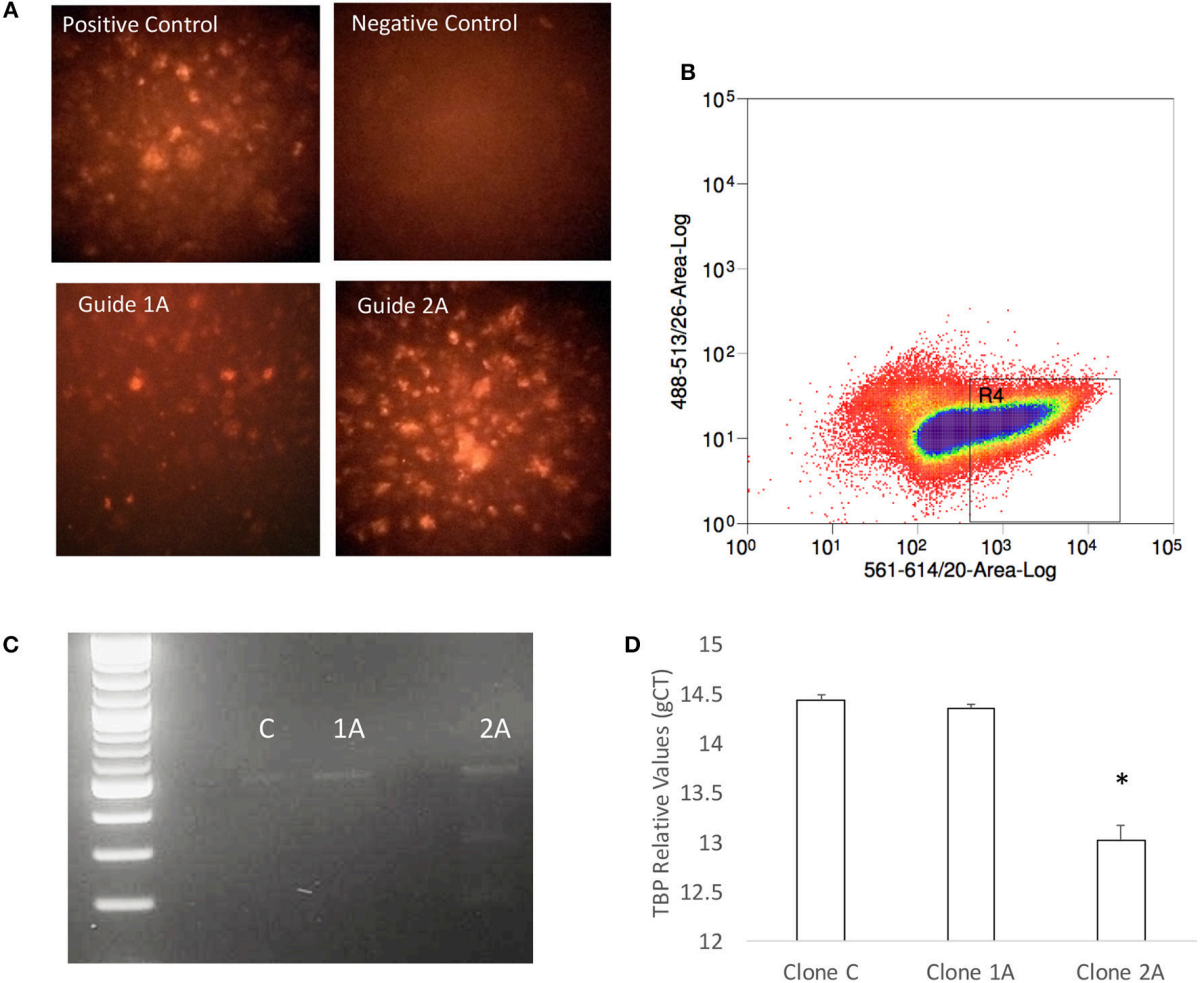
Generation of a TBP-deficient THP1 clone. **(A)** Seventy-two hours after exposure to gesicles containing the TBP guide sequence and Cas9, positively transfected cells were visualized under a fluorescence-microscope for detection of intracellular red fluorescence signal originated from the Cherry Picker reporter on the gesicles' surface. Positive control provided by the manufacturer was compared with a negative control performed with empty gesicles, and two clones that received the guide sequence. Clone 1A showed low efficiency, and clone 2A was highly positive. **(B)** The Cherry Picker-positive cells were gated based on fluorescence intensity, and sorted using a BD FACSJazz (BD Biosciences, San Diego, CA). **(C)** The TBP mutation was confirmed in clone 2A using DNA hybridization with Guide-It Resolvase on a gel-purified genomic TBP sequence, and with **(D)** qRT-PCR. Results are the average ± standard error of 2 experiments performed in triplicate.

The predicted significance of TBP as a transcription factor in Tat stimulated cells but not in Meth, or Meth+Tat, was challenged by the similarities in RNA Pol II recruitment found in all three conditions. Thus, we developed a strategy to test whether TBP is indeed important for the changes caused by HIV Tat, as well as by Meth, and their interactions. Our strategy consisted on impairing the expression of the TBP gene, using CRISPR/Cas9 guides delivered into THP1 cells by using exosomic gesicles. The efficient transfection of guide sequences was monitored using mCherryPicker reporter (Figure 7A). One representative high efficiency clone (2A), was compared to a low efficiency clone (1A) and to a control clone (C) that was treated with empty gesicles. Cells that were positive to mCherryPicker, indicating efficient delivery of guide sequences were sorted using flow cytometry procedures, and placed in culture for expansion and testings (Figure 7B). The disruption of the TBP gene was confirmed using Resolvase and visualized in a 2% gel (Figure 7C). The significantly decreased baseline expression of the TBP gene was confirmed using qRT-PCR (Figure 7D).

The confirmation of the disruption in TBP expression included the examination of potentially remaining splice variants (Table 3), where we observed only residual expression of TBP_4, but complete clearance of TBP_7 splice variant.

**Table 3:** TBP splice variant reads in Control and 2A clones.

**Feature ID**	**Transcript ID**	**Experiment - Range**	**C clone (unique transcript reads)**	**2A clone (unique transcript reads)**	**Kahl's *Z*-test *p* value**
TBP_1	ENST00000421512	1	33	0	0.000703333
TBP_2	ENST00000446829	0	0	0	1
TBP_3	ENST00000616883	12	12	1	0.001138457
TBP_4	ENST00000230354	76	361	24	0.01604091
TBP_5	ENST00000423353	0	0	0	1
TBP_6	ENST00000540980	0	0	0	1
TBP_7	ENST00000392092	143	489	0	0.000164347

*The potential of residual TBP expression through alternative splice was estimated in the Control and 2A clones using RNA seq. The number of reads in both clones and p values are reported*.

**Figure 8:**
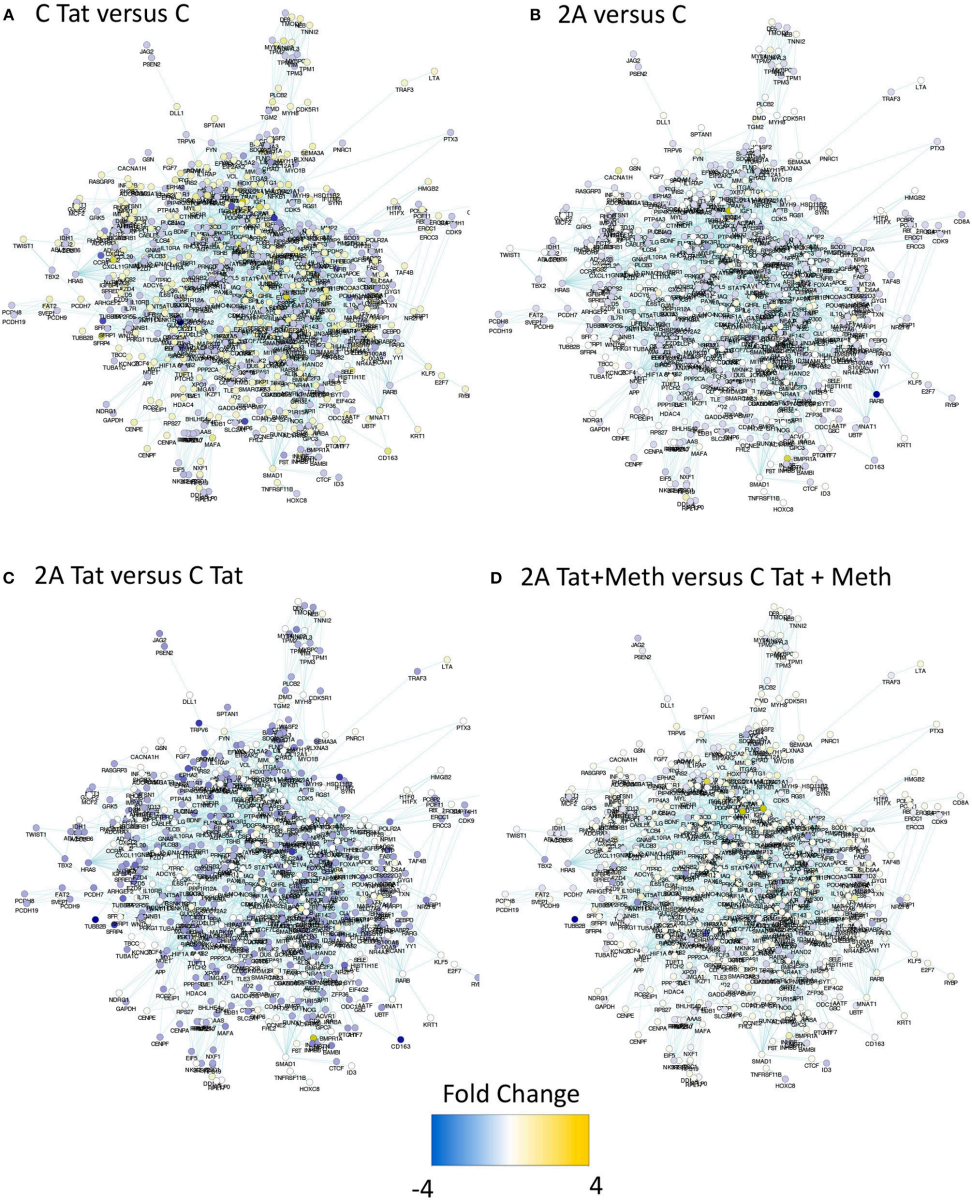
Impact of TBP deficiency on the expression of genes in the TBP targetome following stimulation with Tat, in the presence and in the absence of Meth. The genes that contain Tata box promoter elements and their relationship was estimated in Homo sapiens 3.4 Biogrid default network using iRegulon, with TBP as a factor, and filtered to contain genes that were significantly represented in THP1 cells based on RNA Pol II recruitment capacity at any given experimental condition. The produced mega-network containing 1811 genes was denominated TBP targetome, and used to determine the effect of TBP depletion on Tat stimulation, alone or in the context of Meth. For that, RNA seq was performed in the control clone C and in the TBP-deficient clone 2A, stimulated with 10 μg/ml of HIV Tat, alone or together with 60 μM of Meth. The expression changes were estimated 24 h after stimulation. Yellow tones represent upregulation, and blue tones represent downregulation. **(A)** Clone C treated with Tat was compared to clone C treated with vehicle, showing that the majority of the genes in this network are yellow, indicating that Tat upregulates genes with a TBP binding domain. **(B)** Vehicle-treated clones 2A and C were compared, showing little or no change in color, indicating that they do not differ at baseline. **(C)** Clone 2A stimulated with Tat was compared to clone C stimulated with Tat, showing that most genes are in blue nodes indicating that clone 2A had a lower expression than clone C, upon Tat stimulation. **(D)** Clone 2A treated with Tat+Meth was compared with clone C also treateated with Tat+Meth, where nodes with no color change or yellow indicate that in the presence of Meth, the TBP deficient clone 2A either does not differ from clone C or further increases the expression of genes with a TBP binding promoter domain.

In order to estimate the impact of TBP depletion on the response to HIV-1 Tat, alone and in the context of Meth exposure, a TBP-regulated gene network was identified in the RNA Pol II gene list, with Biogrid Homo sapiens 3.4 gene network. We then used iRegulon (56) in Cytoscape environment, to reverse-engineer the TBP transcriptional regulatory network through the detection of enriched TATA box motifs and their optimal sets of targets in the RNA Pol II data, pooled from all experimental conditions performed in TBP-sufficient clones (Figure 8 and Supplementary Table 1). The resulting TBP targetome contained 1,811 genes identified in THP1 cells. The expression levels of these genes was estimated by RNA seq performed in the control THP1 clones (C), and in the clone that was depleted of TBP gene expression using Crispr/Cas9, upon the stimulation with HIV-1 Tat, or with Tat plus Meth. Figure 8A shows that control C clones stimulated with Tat had a global upregulation of the genes in that network (gene nodes with yellow color), suggesting a significant effect orchestrated through TBP. In Figure 8B, the TBP targetome gene network in the TBP-deficient clone 2A that has either similar (white nodes) or lower baseline expression (blue nodes) when compared to control clone C. Interestingly, the genes in the TBP targetome were significantly suppressed in Tat-stimulated TBP-deficient clone 2A compared to clone C, as revealed by the higher number and intensity of blue colored nodes in the network, and confirming that these genes, under Tat stimulation, were predominantly transcribed via Tata box promoter element, and in a TBP-dependent manner (Figure 8C). However, when Tat was incubated together with Meth, the genes in the TBP targetome were upregulated even in clone 2A, bypassing the TBP deficiency, as shown by the higher number of yellow colored nodes, and suggesting that in the context of Meth exposure, a diversification of transcriptional factors plays a role in activating the same promoters (Figure 8D), as a potential mechanism of exacerbated consequences in HIV and drug abuse.

**Figure 9:**
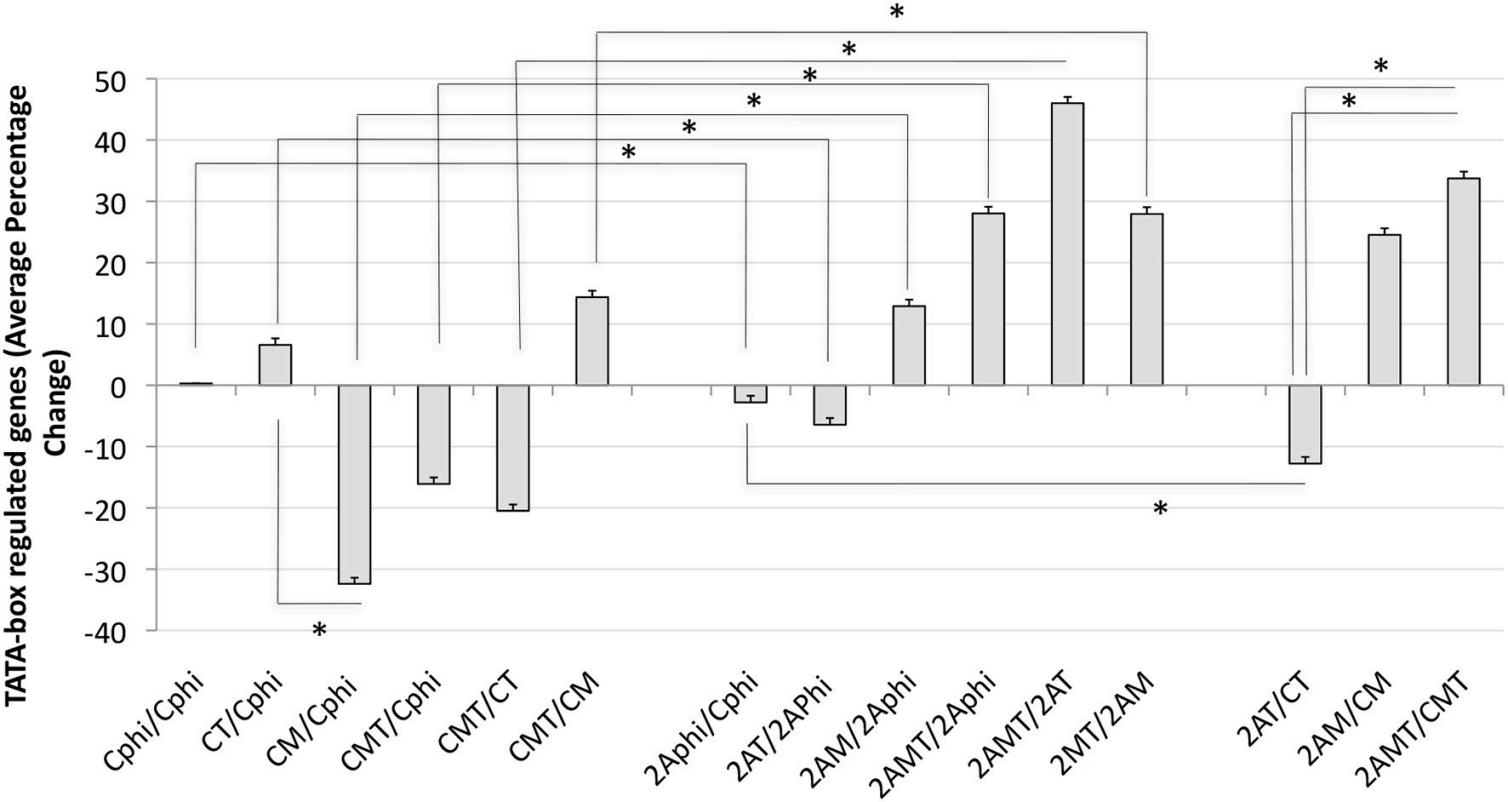
Global changes in the TBP targetome in TBP-sufficient and –deficient monocyte clones after Tat stimulation with or without Meth. The sum of all fold changes in genes that have a Tata box promoter element upon designated stimulations. C is Control clone and 2A is the TBP-defficient clone. T refers to Tat stimulation, M to Meth stimulation, and MT is their combination. Phi refers to control function. Results represent the average ± standard deviation of expression of 1,811 genes included in the TBP megatargetome identified in iRegulon. *p < 0.05 in assigned comparisons, using ANOVA and Bonferroni's *post hoc* test.

In order to further estimate the impact of TBP in Tat stimulation and the effect of Meth, global changes in the TBP targetome genes were calculated by the sum of fold changes in all 1811 genes, in clones C and 2A, upon Tat, plus and minus Meth stimulation (Figure 9). The strategy showns that Tat globally increased TATA-box bearing genes by an average of 8.6%, in the control clone (CT/CPhi), but failed to do it so in the clone 2A (2AT/2APhi). However, Tat in combination with Meth increased the same genes in the TBP-defficient clone 2A by an average of 29% (2AMT/2APhi).

**Figure 10:**
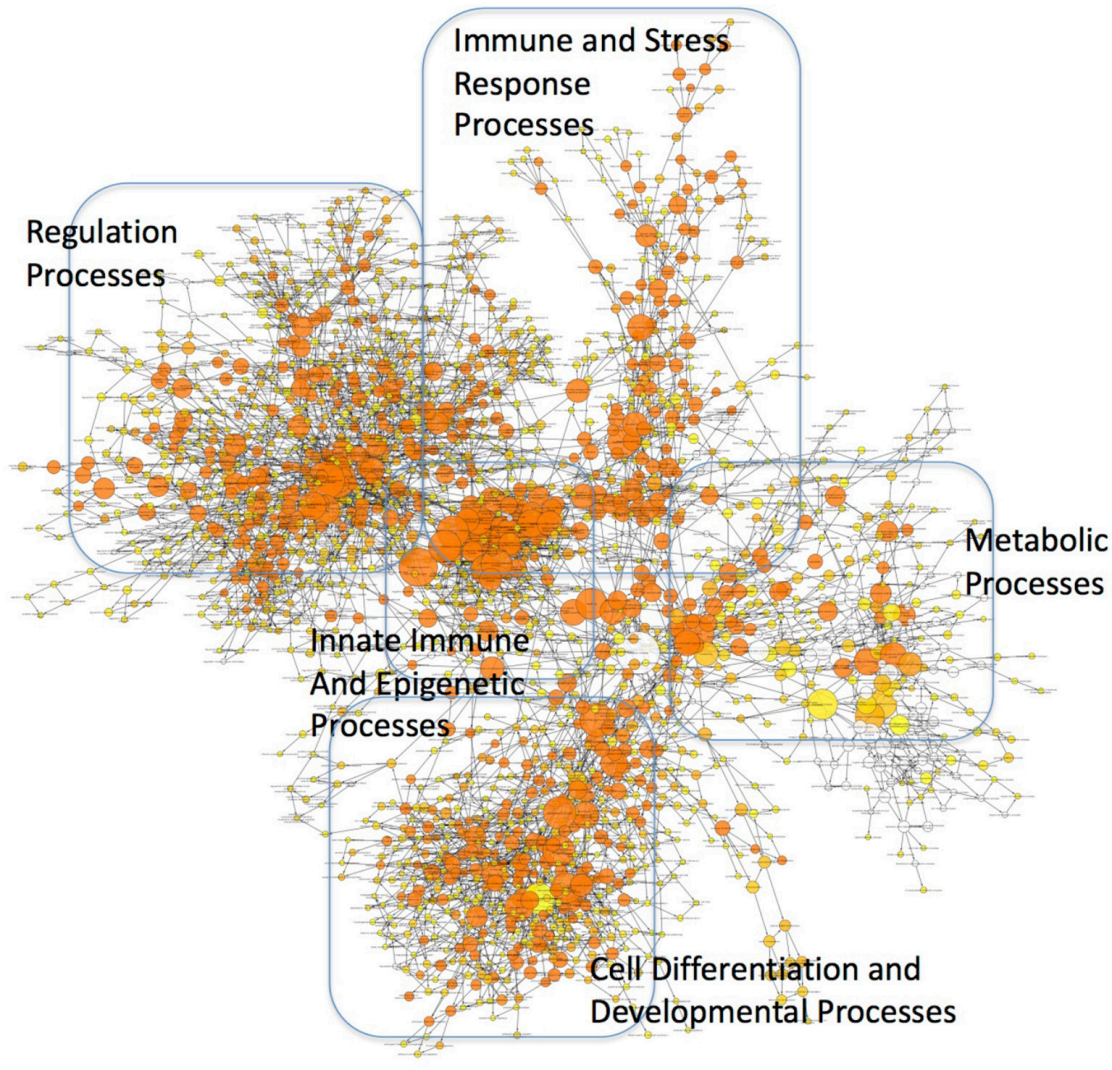
Mapping of functional categories identified in genes of the TBP targetome. Gene Ontology (GO) analysis was performed using BinGO. The circles' size show overrepresentation, and colors report perturbation, orange being high, and yellow, low.

These findings may have profound implications to the character of the inflammatory pathogenesis in HIV-infected Meth abusers. A prediction of the involvement of TBP-regulated genes in molecular pathways was examined using BinGO (57, 58) (Figure 10 and Supplementary Table 2). TBP-regulated genes are predominantly involved in immune and inflammatory pathways, but also in epigenetic, regulatory and metabolic pathways. In Figure 10, large circles report overrepresentation, and orange color report higher perturbation, to allow the visualization of stronger effects on innate immune and epigenetic function, as well as regulation (adjusted *p* = 0.00136, *p* = 0.000601, respectively). Within the whole TBP targetome, genes that failed to be upregulated in clone 2A upon Tat stimulation were functionally associated to the nucleus, DNA-binding, transcription, nucleotide binding, developmental protein and cytoskeleton (Benjamini *p* < 0.01), but genes involved in tyrosine-protein kinase, Wnt-signaling, ATP binding, methylation and tight junction were also represented (Benjamini *p* < 0.05). Main identified TBP-controlled pathways using this strategy were assessed in KEGG, and are shown in Table 4.

**Table 4:** KEGG pathway analysis of genes that failed to upregulate in response to Tat in TBP-deficient 2A clones.

**Genes Pathway**	***p* value**	**Benjamini**
Proteoglycans in cancer	0.00043	0.067
Hedgehog signaling pathway	0.00076	0.059
Axon guidance	0.001	0.052
Signaling pathways regulating pluripotency of stem cells	0.0018	0.07
Central carbon metabolism in cancer	0.0038	0.11
FoxO signaling pathway	0.0052	0.13
Transcriptional misregulation in cancer	0.0067	0.14
HTLV-I infection	0.01	0.18
Basal cell carcinoma	0.012	0.2
Tight junction	0.026	0.34
Wnt signaling pathway	0.026	0.34
Pathways in cancer	0.027	0.32
Hippo signaling pathway	0.039	0.41
Mineral absorption	0.043	0.42
AMPK signaling pathway	0.046	0.42
Notch signaling pathway	0.048	0.41
Progesterone-mediated oocyte maturation	0.05	0.4

**Figure 11:**
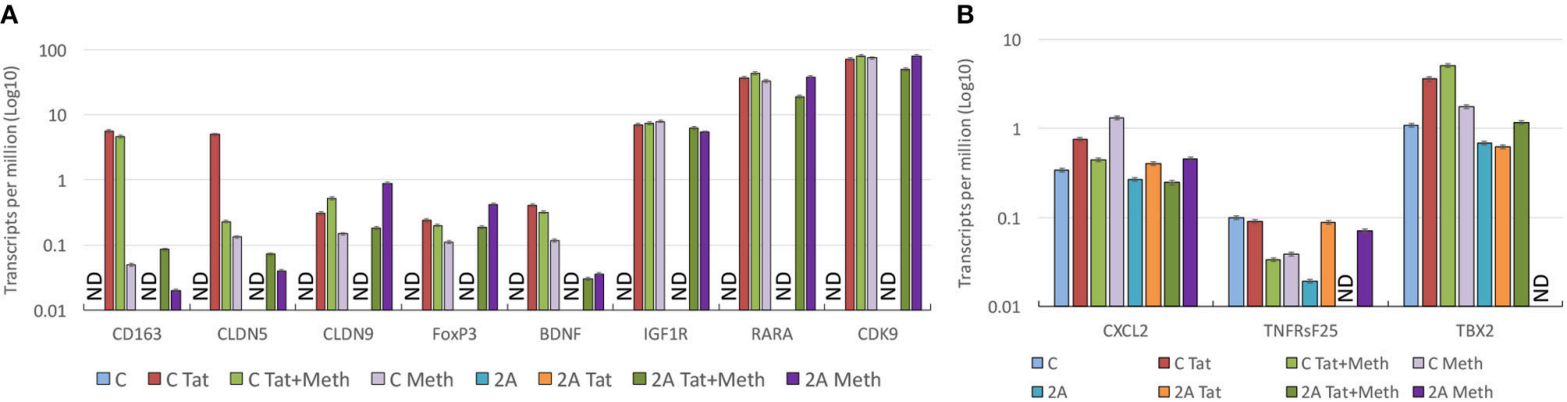
Effect of TBP transcription factor in the upregulation of gene transcripts in response to Tat and Meth. Gene transcript counts are represented in log10 of transcripts per million (TPM). The axis in log allows the appreciation of genes that are increased with different intensities for comparison of their behaviors in response to stimulation. **(A)** Representative TBP targets illustrating the changes in genes that are undetectable in unstimulated cells and responsive to Tat and/or Meth. **(B)** Representative hits illustrating the behavior of genes that are constitutively transcribed in unstimulated cells, and not responsive to Tat and/or Meth. ND, Not detectable.

Genes that play a role in inflammatory pathology, and that have been described to have an involvement in HIV pathogenesis, in the brain and elsewhere, were selected for a closer validation of the role of TBP in Tat stimulation, and of the ability of Meth as a co-morbidity to enhance those same genes through other transcription factors. A detailed examination of genes that have a TATA box promoter element and their changes in response to Tat in the TBP-sufficient (C) and deficient (2A) clones reveals two classes of genes, which seem to segregate based on their expression levels in unstimulated controls (Figure 11). The genes in Figure 11A, which are not detectable in the unstimulated clone C or clone 2A, are highly induced by Tat in clone C, but not in clone 2A, suggesting that the transcription of these genes upon HIV-1 Tat stimulation is primarily triggered via the TATA box promoter domain, under TBP control. On the other hand, the stimulation with Tat in the presence of Meth, or Meth alone, was able to increase these transcripts in both clones, and bypassing the TBP deficiency in clone 2A, confirming a diversification of transcription factor usage by the drug, alone or in the context of HIV Tat. Examples include genes involved in blood brain barrier permeability, inflammation and immune regulation, neuroprotection and metabolic outcomes, such as CD163, Claudins 5 and 9 (CLDN5 and CLDN9), FoxP3, Brain-derived neurotrophic factor (BDNF), Insulin growth factor 1 Receptor (IGF1R), Retinoic acid receptor alpha (RARA), and the important kinase CDK9. The genes in Figure 10B also present TATA box promoter element, and were detected in the TBP targetome, but differed from the genes represented in Figure 11A by being constitutively expressed in unstimulated cells, regardless of the TBP expression. Interestingly, these genes were not responsive to Tat, or to Meth. This was the case for instance of CXCL2, Tumor necrosis factor receptor superfamily member 25 (TNFRsF25), and the T-box 2 transcription factor (TBX2).

Overall, our results indicate that the HIV-1 Tat peptide acts through the usage of TBP as a transcription factor that engages in the Tata-box binding motif in the promoter of promptly inducible genes, which are expressed at low or undetectable levels in unstimulated resting states. These genes include a large collection of inflammatory genes, regulatory elements and metabolic components. In the presence of Meth, which is a common comorbidity of HIV infection, a larger number of transcription factors may increase the probability of transcription, and increase the number of transcribed early response genes, explaining the exacerbation of pathogenic hallmarks, particularly in the brain of HIV+ drug users.

## Discussion

The role of the Tata-box promoter domain in HIV transcription has been suggested (23). In addition, single nucleotide polymorphisms in its sequence located in the long-terminal repeat (LTR) in the viral transcription complex, which confers a low affinity to TBP, the Tata-box binding transcription factor, results in significantly slower replication (59). The main HIV-1 peptide that is responsible for the transactivation of viral transcription, Tat, performs its task by directly inducing and recruiting TBP to the LTR, via its ability to directly interact with the cellular cofactor positive transcription elongation factor b (P-TEFb), which bridges TBP binding to the promoter without the need of other TBP-associated factors (23). While this is a mechanism that is relevant to promote viral replication, here we investigated the hypothesis that HIV-1 Tat is able to activate transcription of thousands of host genes through a TBP-dependent strategy, producing hyper-inflammatory phenotypes that are further beneficial to the viral propagation.

By engineering a macrophage cell line that is deficient on the expression of dominant TBP splice variants, we demonstrated that transcriptional changes induced by the incubation with HIV-1 Tat favor inducible genes that have the Tata-box promoter sequence in their promoters. We also showed that such effect is achieved through the dominant usage of TBP as a transcription factor. Other transcription factors that may be triggered, cJun, interferon regulatory factor 2 (IRF2), cFos and POU6F1, also appear to be triggered, as their binding motifs are identified in the promoters of genes with increased RNA Pol recruitment, and can be controlling different sets of genes. These findings may have important implications for understanding mechanisms of pathogenesis in HIV infection, and potentially for therapy. We also showed that the exposure of macrophages to a stimulant drug, Meth, together with HIV-1 Tat, is able to activate the same gene promoters, which is revealed by the efficient recruitment of RNA Pol. However, this happens through a distinctive and more diversified usage of transcriptional factors. The absence of TAF9 in Meth-stimulated cells, and the results of the analysis of consensus promoter sequences in TBP-sufficient cells stimulated with Tat plus Meth, support that an alternative to TBP is triggered by Meth, it can potentially co-exist with TBP in the context of HIV-1 Tat, but ultimately support promoter activation through different motifs. This may suggest that co-morbidities represent a factor of complexity in therapies that target transcriptional machineries.

We have shown that TBP and Tat are translocated within 5 min into the cell nucleus, when in the presence of Meth, which may have important implications to the viral transcription. However, we did not test the effects of TBP disruption on viral transcription or replication, but exclusively on the effects of Tat as a factor that enhances transcription of host genes. The identification of TBP as a the most frequently aligned promoter motif in genes with RNA Pol efficiently recruited, suggested that TBP played an important role. Yet, it does not explain the basis for the interaction between Tat and TBP. Further experiments are necessary to establish the molecular basis for that interaction in the context of absence of TAR, or on the effects over the LTR. Although TBP was the most frequent transcription factor motif, other transcription motifs were also significant, such as cJun/cFos, IRF2, and POU6F1. In other systems, Sp-1 has been suggested as a Tat molecular partner, bridged by Cyclin T1/CDK9 complexes, which can promote transcription of the virus in a TAR-independent fashion (60–62), and likely also of host gene promoters. Sp-1 did not appear as a binding motif in Tat-enhanced promoters, but it was one of the factors associated with Meth-induced activation. Thus, in the presence of Meth, or in the absence of TBP, transcription factor redundancy and promoter plasticity may perpetuate and broaden gene transcription, including of HIV.

Two transcription factors appeared to be common between HIV-1 Tat and Meth, which were Egr-1, and IRF4. Further studies are necessary to examine which common genes can be controlled by these transcription factors, to estimate their value as targets of intervention. In the non-human primate model of neuroHIV, we have previously demonstrated that a decrease in Egr-1 expression in neurons is associated with neurological deficits in the context of inflammation (63). Its role in cell transcriptional regulation has been described (64). IRF4, on the other hand, is a positive regulator of inflammation (65, 66), converge as a common epigenetic switch.

Our results suggest that the occurrence of additional cellular stimulus in HIV-1 may deviate transcription factor usage, by promoting diversity. However, a role for TBP inhibitors should not be ruled out as a strategy to ameliorate stimulant drug users. For instance, it has been suggested that although the Tata-box promoter domain sequences are selectively used by TBP *in vivo*, the binding can be modulated by TBP inhibitors such as TBA-associated factor 1 (TAF1) (67–69). In addition, Tat in combination with Meth enhanced the acute translocation of Tat into the cellular nucleus, which may have implications to the viral transcription, not examined here.

Overall, we have found a key role for TBP in the induction of genes containing the TATA-box core promoter element by the HIV Tat peptide, characterized by early response, inflammatory and metabolic genes. We have also determined the effect of the interactions between Tat and Meth, a common co-morbidity of HIV infection, on diversifying the collection of transcription factors able to redundantly act on the activation of promoters, RNA Pol recruitment and ultimately transcription of genes with important implications in inflammatory pathology. Our findings help explain aggravated phenotypes associated to inflammation and to metabolic disorders that are commonly observed in HIV+ individuals with substance use disorders, but also suggest that in these individuals, targeting elements of the transcriptional machinery posits the risk of failure, while dealing with additional molecular complexities.

## Author Contributions

RT performed the Crispr/Cas9 disruption of TBP expression, tested and selected the positive clones, and performed all the *in vitro* stimulations. JAN performed the fixation of cells for RNA Pol ChIP assay. AL performed THP1-deficient cells PCRs. LAC provided guidance for the design and execution of Crispr/Cas9-based TBP disruption, reviewed all the results, helped in the analysis, and in the writing of the manuscript. JJ tested all the primers for PCR validation. LB participated in the experimental design, performed all TBP-sufficient cell cultures and stimulation experiments, fixations for ChIP assays, performed PCRs, and helped in writing the manuscript. MM developed the concept, designed the study, obtained funding, supervised experiments, menthored students, revised all results, performed all systems biology-based approaches, revised statistical results, and wrote the manuscript.

### Conflict of Interest Statement

The authors declare that the research was conducted in the absence of any commercial or financial relationships that could be construed as a potential conflict of interest.

## Supplementary Material

Supplementary Table 1 TBP megatargetome-RNA Pol ChIP average peak values in genes that have a TATA-box binding motif in the core promoter element. Cross linking for RNA Pol II ChIP was performed 15 min after stimulation with HIV Tat (10 ng/ml), Meth (60 μM), or Tat+Meth, for determination of gene intervals, and average peak ratios.

Supplementary Table 2 The effect of TBP disruption in the response to Tat and/or Meth. RNA-seq was performed in TBP-defficient clone 2A and compared to TBP-sufficient clone C. T = Tat stimulation, M = Meth stimulation, MT = Meth+Tat. Gene IDs, Transcript ID, chromosomes and gene intervals are provided. This experiment was performed in triplicate.

Supplementary Figure S1 TBP nuclear translocation. The PMA-differentiated THP1 cells were stimulated with Meth (60 μM), and/or HIV Tat (10 ng/ml) for examination of TBP nuclear translocation 5, 15, 30, and 60 min following stimulation, by immunocytochemistry. TBP was stained in red, and nuclei were visualized with DAPI (blue). Confocal images were used for the determination of a translocation index, as the percentage of total TBP staining that was co-localized with DAPI. Representative images of **(A)** Controls and Tat stimulation, **(B)** Tat+Meth stimulation. This experiment was performed three times in triplicate.

## References

[1] Basova L Najera JA Bortell N Wang D Moya R Lindsey A. Dopamine and its receptors play a role in the modulation of CCR5 expression in innate immune cells following exposure to Methamphetamine: implications to HIV infection. PLoS ONE (2018) 13:e0199861. 10.1371/journal.pone.0199861 29944719 PMC6019408

[2] Bosshart H Heinzelmann M. THP-1 cells as a model for human monocytes. Ann Transl Med. (2016) 4:438. 10.21037/atm.2016.08.53 27942529 PMC5124613

[3] Fauci AS. Host factors and the pathogenesis of HIV-induced disease. Nature (1996) 384:529–34. 10.1038/384529a0 8955267

[4] Lindl KA Marks DR Kolson DL Jordan-Sciutto KL. HIV-associated neurocognitive disorder: pathogenesis and therapeutic opportunities. J Neuroimmune Pharmacol. (2010) 5:294–309. 10.1007/s11481-010-9205-z 20396973 PMC2914283

[5] Marcondes MC Flynn C Watry DD Zandonatti M Fox HS. Methamphetamine increases brain viral load and activates natural killer cells in simian immunodeficiency virus-infected monkeys. Am J Pathol. (2010) 177:355–61. 10.2353/ajpath.2010.090953 20489154 PMC2893678

[6] Nath A Maragos WF Avison MJ Schmitt FA Berger JR. Acceleration of HIV dementia with methamphetamine and cocaine. J Neurovirol. (2001) 7:66–71. 10.1080/135502801300069737 11519485

[7] Najera JA Bustamante EA Bortell N Morsey B Fox HS Ravasi T. Methamphetamine abuse affects gene expression in brain-derived microglia of SIV-infected macaques to enhance inflammation and promote virus targets. BMC Immunol. (2016) 17:7. 10.1186/s12865-016-0145-0 27107567 PMC4841970

[8] Bortell N Morsey B Basova L Fox HS Marcondes MC. Phenotypic changes in the brain of SIV-infected macaques exposed to methamphetamine parallel macrophage activation patterns induced by the common gamma-chain cytokine system. Front Microbiol. (2015) 6:900. 10.3389/fmicb.2015.00900 26441851 PMC4568411

[9] Debaisieux S Rayne F Yezid H Beaumelle B. The ins and outs of HIV-1 Tat. Traffic (2012) 13:355–63. 10.1111/j.1600-0854.2011.01286.x 21951552

[10] Rayne F Debaisieux S Bonhoure A Beaumelle B. HIV-1 Tat is unconventionally secreted through the plasma membrane. Cell Biol Int. (2010) 34:409–13. 10.1042/CBI20090376 19995346

[11] Rayne F Debaisieux S Tu A Chopard C Tryoen-Toth P Beaumelle B Detecting HIV-1 Tat in cell culture supernatants by ELISA or Western Blot. Methods Mol Biol. (2016) 1354:329–42. 10.1007/978-1-4939-3046-3_22 26714722

[12] Vendeville Rayne F Bonhoure A Bettache N Montcourrier P Beaumelle B. HIV-1 Tat enters T cells using coated pits before translocating from acidified endosomes and eliciting biological responses. Mol Biol Cell (2004) 15:2347–60. 10.1091/mbc.e03-12-0921 15020715 PMC404028

[13] Carey AN Liu X Mintzopoulos D Paris JJ Muschamp JW McLaughlin JP. Conditional Tat protein expression in the GT-tg bigenic mouse brain induces gray matter density reductions. Prog Neuro Psychopharmacol Biol Psychiatry (2013) 43:49–54. 10.1016/j.pnpbp.2012.12.018 23269344 PMC3612135

[14] Carey AN Sypek EI Singh HD Kaufman MJ McLaughlin JP. Expression of HIV-Tat protein is associated with learning and memory deficits in the mouse. Behav Brain Res. (2012) 229:48–56. 10.1016/j.bbr.2011.12.019 22197678 PMC3580389

[15] Kesby JP Najera JA Romoli B Fang Y Basova L Birmingham A. HIV-1 TAT protein enhances sensitization to methamphetamine by affecting dopaminergic function. Brain Behav Immun. (2017) 65:210–21. 10.1016/j.bbi.2017.05.004 28495611 PMC5537017

[16] Bannwarth S Gatignol A. HIV-1 TAR RNA: the target of molecular interactions between the virus and its host. Curr HIV Res. (2005) 3:61–71. 10.2174/1570162052772924 15638724

[17] Brady J Kashanchi F. Tat gets the “green” light on transcription initiation. Retrovirology (2005) 2:69. 10.1186/1742-4690-2-69 16280076 PMC1308864

[18] Davidson A Leeper TC Athanassiou Z Patora-Komisarska K Karn J Robinson JA. Simultaneous recognition of HIV-1 TAR RNA bulge and loop sequences by cyclic peptide mimics of Tat protein. Proc Natl Acad Sci USA. (2009) 106:11931–6. 10.1073/pnas.0900629106 19584251 PMC2715490

[19] Naryshkin NA Gait MJ Ivanovskaya MG. RNA recognition and regulation of HIV-1 gene expression by viral factor Tat. Biochemistry (1998) 63:489–503. 9632883

[20] Richter Ping YH Rana TM. TAR RNA loop: a scaffold for the assembly of a regulatory switch in HIV replication. Proc Natl Acad Sci USA. (2002) 99:7928–33. 10.1073/pnas.122119999 12048247 PMC122997

[21] Wei P Garber ME Fang SM Fischer WH Jones KA. A novel CDK9-associated C-type cyclin interacts directly with HIV-1 Tat and mediates its high-affinity, loop-specific binding to TAR RNA. Cell (1998) 92:451–62. 10.1016/S0092-8674(00)80939-3 9491887

[22] Bieniasz PD Grdina TA Bogerd HP Cullen BR. Recruitment of cyclin T1/P-TEFb to an HIV type 1 long terminal repeat promoter proximal RNA target is both necessary and sufficient for full activation of transcription. Proc Natl Acad Sci USA. (1999) 96:7791–6. 10.1073/pnas.96.14.7791 10393900 PMC22140

[23] Raha T Cheng SW Green MR. HIV-1 Tat stimulates transcription complex assembly through recruitment of TBP in the absence of TAFs. PLoS Biol. (2005) 3:e44. 10.1371/journal.pbio.0030044 15719058 PMC546330

[24] Verrijzer CP Yokomori K Chen JL Tjian R. Drosophila TAFII150: similarity to yeast gene TSM-1 and specific binding to core promoter DNA. Science (1994) 264:933–41. 10.1126/science.8178153 8178153

[25] Kim YS Panganiban AT. Examination of TAR-independent Trans activation by human immunodeficiency virus type 1 Tat in human glial cells. J Neurosci Res. (1996) 43:652–66. 10.1002/(SICI)1097-4547(19960315)43:6<652::AID-JNR2>3.0.CO;2-D 8984195

[26] Pu H Tian J Flora G Lee YW Nath A Hennig B. HIV-1 Tat protein upregulates inflammatory mediators and induces monocyte invasion into the brain. Mol Cell Neurosci. (2003) 24:224–37. 10.1016/S1044-7431(03)00171-4 14550782

[27] Toborek M Lee YW Pu H Malecki A Flora G Garrido R. HIV-Tat protein induces oxidative and inflammatory pathways in brain endothelium. J Neurochem. (2003) 84:169–79. 10.1046/j.1471-4159.2003.01543.x 12485413

[28] Roebuck KA Rabbi MF Kagnoff MF. HIV-1 Tat protein can transactivate a heterologous TATAA element independent of viral promoter sequences and the trans-activation response element. AIDS (1997) 11:139–46. 10.1097/00002030-199702000-00002 9030359

[29] Veschambre P Simard P Jalinot P. Evidence for functional interaction between the HIV-1 Tat transactivator and the TATA box binding protein in vivo. J Mol Biol. (1995) 250:169–80. 10.1006/jmbi.1995.0368 7608968

[30] Andras IE Pu H Deli MA Nath A Hennig B Toborek M. HIV-1 Tat protein alters tight junction protein expression and distribution in cultured brain endothelial cells. J Neurosci Res. (2003) 74:255–65. 10.1002/jnr.10762 14515355

[31] Ben Haij N Planes R Leghmari K Serrero M Delobel P Izopet J HIV-1 Tat protein induces production of proinflammatory cytokines by human dendritic cells and monocytes/macrophages through engagement of TLR4-MD2-CD14 complex and activation of NF-kappaB pathway. PLoS ONE (2015) 10:e0129425 10.1371/journal.pone.0129425 26090662 PMC4474861

[32] Ben Haij N Leghmari K Planes R Thieblemont N Bahraoui E HIV-1 Tat protein binds to TLR4-MD2 and signals to induce TNF-alpha and IL-10. Retrovirology (2013) 10:123 10.1186/1742-4690-10-123 24165011 PMC4231456

[33] Leghmari K Bennasser Y Bahraoui E. HIV-1 Tat protein induces IL-10 production in monocytes by classical and alternative NF-kappaB pathways. Eur J Cell Biol. (2008) 87:947–62. 10.1016/j.ejcb.2008.06.005 18760861

[34] Contreras X Bennasser Y Bahraoui E. IL-10 production induced by HIV-1 Tat stimulation of human monocytes is dependent on the activation of PKC beta(II) and delta isozymes. Microbes Infect. (2004) 6:1182–90. 10.1016/j.micinf.2004.06.008 15488737

[35] Bennasser Y Badou A Tkaczuk J Bahraoui E. Signaling pathways triggered by HIV-1 Tat in human monocytes to induce TNF-alpha. Virology (2002) 303:174–80. 10.1006/viro.2002.1676 12482669

[36] Bennasser Y Contreras X Moreau M Le Clerc C Badou A Bahraoui E. HIV-1 Tat protein induces IL-10 production by human monocytes: implications of the PKC and calcium pathway. J Soc Biol. (2001) 195:319–26. 10.1051/jbio/2001195030319 11833470

[37] Vives E Charneau P van Rietschoten J Rochat H Bahraoui E. Effects of the Tat basic domain on human immunodeficiency virus type 1 transactivation, using chemically synthesized Tat protein and Tat peptides. J Virol. (1994) 68:3343–53. 8151793 10.1128/jvi.68.5.3343-3353.1994 PMC236825

[38] Hahn S Buratowski S Sharp PA Guarente L. Isolation of the gene encoding the yeast TATA binding protein TFIID: a gene identical to the SPT15 suppressor of Ty element insertions. Cell (1989) 58:1173–81. 10.1016/0092-8674(89)90515-1 2550146

[39] Hahn S Buratowski S Sharp PA Guarente L. Yeast TATA-binding protein TFIID binds to TATA elements with both consensus and nonconsensus DNA sequences. Proc Natl Acad Sci US A. (1989) 86:5718–22. 10.1073/pnas.86.15.5718 2569738 PMC297701

[40] Yang C Bolotin E Jiang T Sladek FM Martinez E. Prevalence of the initiator over the TATA box in human and yeast genes and identification of DNA motifs enriched in human TATA-less core promoters. Gene (2007) 389:52–65. 10.1016/j.gene.2006.09.029 17123746 PMC1955227

[41] Suzuki Y Tsunoda T Sese J Taira H Mizushima-Sugano J Hata H. Identification and characterization of the potential promoter regions of 1031 kinds of human genes. Genome Res. (2001) 11:677–84. 10.1101/gr.GR-1640R 11337467 PMC311086

[42] Tullai JW Schaffer ME Mullenbrock S Sholder G Kasif S Cooper GM. Immediate-early and delayed primary response genes are distinct in function and genomic architecture. J Biol Chem. (2007) 282:23981–95. 10.1074/jbc.M702044200 17575275 PMC2039722

[43] Marban C Su T Ferrari R Li B Vatakis D Pellegrini M. Genome-wide binding map of the HIV-1 Tat protein to the human genome. PLoS ONE (2011) 6:e26894. 10.1371/journal.pone.0026894 22073215 PMC3208564

[44] Reeder JE Kwak YT McNamara RP Forst CV D'Orso I. HIV Tat controls RNA Polymerase II and the epigenetic landscape to transcriptionally reprogram target immune cells. Elife (2015) 4:e08955. 10.7554/eLife.08955 26488441 PMC4733046

[45] Kashanchi F Piras G Radonovich MF Duvall JF Fattaey A Chiang CM. Direct interaction of human TFIID with the HIV-1 transactivator tat. Nature (1994) 367:295–9. 10.1038/367295a0 8121496

[46] Majello B Napolitano G Lania L. Recruitment of the TATA-binding protein to the HIV-1 promoter is a limiting step for Tat transactivation. AIDS (1998) 12:1957–64. 10.1097/00002030-199815000-00006 9814863

[47] Wilson JM Kalasinsky KS Levey AI Bergeron C Reiber G Anthony RM. Striatal dopamine nerve terminal markers in human, chronic methamphetamine users. Nat Med. (1996) 2:699–703. 10.1038/nm0696-699 8640565

[48] Xu S Grullon S Ge K Peng W. Spatial clustering for identification of ChIP-enriched regions (SICER) to map regions of histone methylation patterns in embryonic stem cells. Methods Mol Biol. (2014) 1150:97–111. 10.1007/978-1-4939-0512-6_5 24743992 PMC4152844

[49] Chekmenev DS Haid C Kel AE. P-Match: transcription factor binding site search by combining patterns and weight matrices. Nucleic Acids Res. (2005) 33:W432–7. 10.1093/nar/gki441 15980505 PMC1160202

[50] Kel AE Gossling E Reuter I Cheremushkin E Kel-Margoulis OV Wingender E. MATCH: A tool for searching transcription factor binding sites in DNA sequences. Nucleic Acids Res. (2003) 31:3576–9. 10.1093/nar/gkg585 12824369 PMC169193

[51] Zuris JA Thompson DB Shu Y Guilinger JP Bessen JL Hu JH. Cationic lipid-mediated delivery of proteins enables efficient protein-based genome editing *in vitro* and *in vivo*. Nat Biotechnol. (2015) 33:73–80. 10.1038/nbt.3081 25357182 PMC4289409

[52] Fu Y Foden JA Khayter C Maeder ML Reyon D Joung JK. High-frequency off-target mutagenesis induced by CRISPR-Cas nucleases in human cells. Nat Biotechnol. (2013) 31:822–6. 10.1038/nbt.2623 23792628 PMC3773023

[53] Lin S Staahl BT Alla RK Doudna JA. Enhanced homology-directed human genome engineering by controlled timing of CRISPR/Cas9 delivery. Elife (2014) 3:e04766. 10.7554/eLife.04766 25497837 PMC4383097

[54] Mali P Aach J Stranges PB Esvelt KM Moosburner M Kosuri S. CAS9 transcriptional activators for target specificity screening and paired nickases for cooperative genome engineering. Nat Biotechnol. (2013) 31:833–8. 10.1038/nbt.2675 23907171 PMC3818127

[55] Hsu PD Scott DA Weinstein JA Ran FA Konermann S Agarwala V. DNA targeting specificity of RNA-guided Cas9 nucleases. Nat Biotechnol. (2013) 31:827–32. 10.1038/nbt.2647 23873081 PMC3969858

[56] Janky R Verfaillie A Imrichova H Van de Sande B Standaert L Christiaens V. iRegulon: from a gene list to a gene regulatory network using large motif and track collections. PLoS Comput Biol. (2014) 10:e1003731. 10.1371/journal.pcbi.1003731 25058159 PMC4109854

[57] Blake JA Harris MA The Gene Ontology (GO) project: structured vocabularies for molecular biology and their application to genome and expression analysis. Curr Protoc Bioinformatics (2002) Chapter 7:Unit 7.2. 10.1002/0471250953.bi0702s23 18792943

[58] Harris MA Clark J Ireland A Lomax J Ashburner M Foulger R. The Gene Ontology (GO) database and informatics resource. Nucleic Acids Res. (2004) 32:D258–61. 10.1093/nar/gkh036 14681407 PMC308770

[59] Suslov VV Ponomarenko PM Efimov VM Savinkova LK Ponomarenko MP Kolchanov NA. SNPs in the HIV-1 TATA box and the AIDS pandemic. J Bioinform Comput Biol. (2010) 8:607–25. 10.1142/S0219720010004677 20556865

[60] Berkhout B Gatignol A Rabson AB Jeang KT. TAR-independent activation of the HIV-1 LTR: evidence that tat requires specific regions of the promoter. Cell (1990) 62:757–67. 10.1016/0092-8674(90)90120-4 2201451

[61] Loregian A Bortolozzo K Boso S Sapino B Betti M Biasolo MA The Sp1 transcription factor does not directly interact with the HIV-1 Tat protein. J Cell Physiol. (2003) 196:251–7. 10.1002/jcp.10271 12811817

[62] Loregian A Bortolozzo K Boso S Caputo A Palu G. Interaction of Sp1 transcription factor with HIV-1 Tat protein: looking for cellular partners. FEBS Lett. (2003) 543:l61–5. 10.1016/S0014-5793(03)00399-5 12753906

[63] Gersten M Alirezaei M Marcondes MC Flynn C Ravasi T Ideker T. An integrated systems analysis implicates EGR1 downregulation in simian immunodeficiency virus encephalitis-induced neural dysfunction. J Neurosci. (2009) 29:12467–76. 10.1523/JNEUROSCI.3180-09.2009 19812322 PMC2802851

[64] Sarkar R Verma SC. Egr-1 regulates RTA transcription through a cooperative involvement of transcriptional regulators. Oncotarget (2017) 8:91425–44. 10.18632/oncotarget.20648 29207655 PMC5710935

[65] Achuthan A Cook AD Lee MC Saleh R Khiew HW Chang MW. Granulocyte macrophage colony-stimulating factor induces CCL17 production via IRF4 to mediate inflammation. J Clin Invest. (2016) 126:3453–66. 10.1172/JCI87828 27525438 PMC5004969

[66] Lech M Weidenbusch M Kulkarni OP Ryu M Darisipudi MN Susanti HE. IRF4 deficiency abrogates lupus nephritis despite enhancing systemic cytokine production. J Am Soc Nephrol. (2011) 22:1443–52. 10.1681/ASN.2010121260 21742731 PMC3148699

[67] Basehoar AD Zanton SJ Pugh BF. Identification and distinct regulation of yeast TATA box-containing genes. Cell (2004) 116:699–709. 10.1016/S0092-8674(04)00205-3 15006352

[68] Chitikila Huisinga KL Irvin JD Basehoar AD Pugh BF. Interplay of TBP inhibitors in global transcriptional control. Mol Cell (2002) 10:871–82. 10.1016/S1097-2765(02)00683-4 12419230

[69] Jackson-Fisher AJ Chitikila C Mitra M Pugh BF. A role for TBP dimerization in preventing unregulated gene expression. Mol Cell (1999) 3:717–27. 10.1016/S1097-2765(01)80004-6 10394360

