# Supplementary figures and images for: Modeling the Function of TATA Box Binding Protein in Transcriptional Changes Induced by HIV-1 Tat in Innate Immune Cells and the Effect of Methamphetamine Exposure
Source: Front Immunol. 2019 Feb 4;9:3110. doi: 10.3389/fimmu.2018.03110 (PMC6369711; doi:10.3389/fimmu.2018.03110)

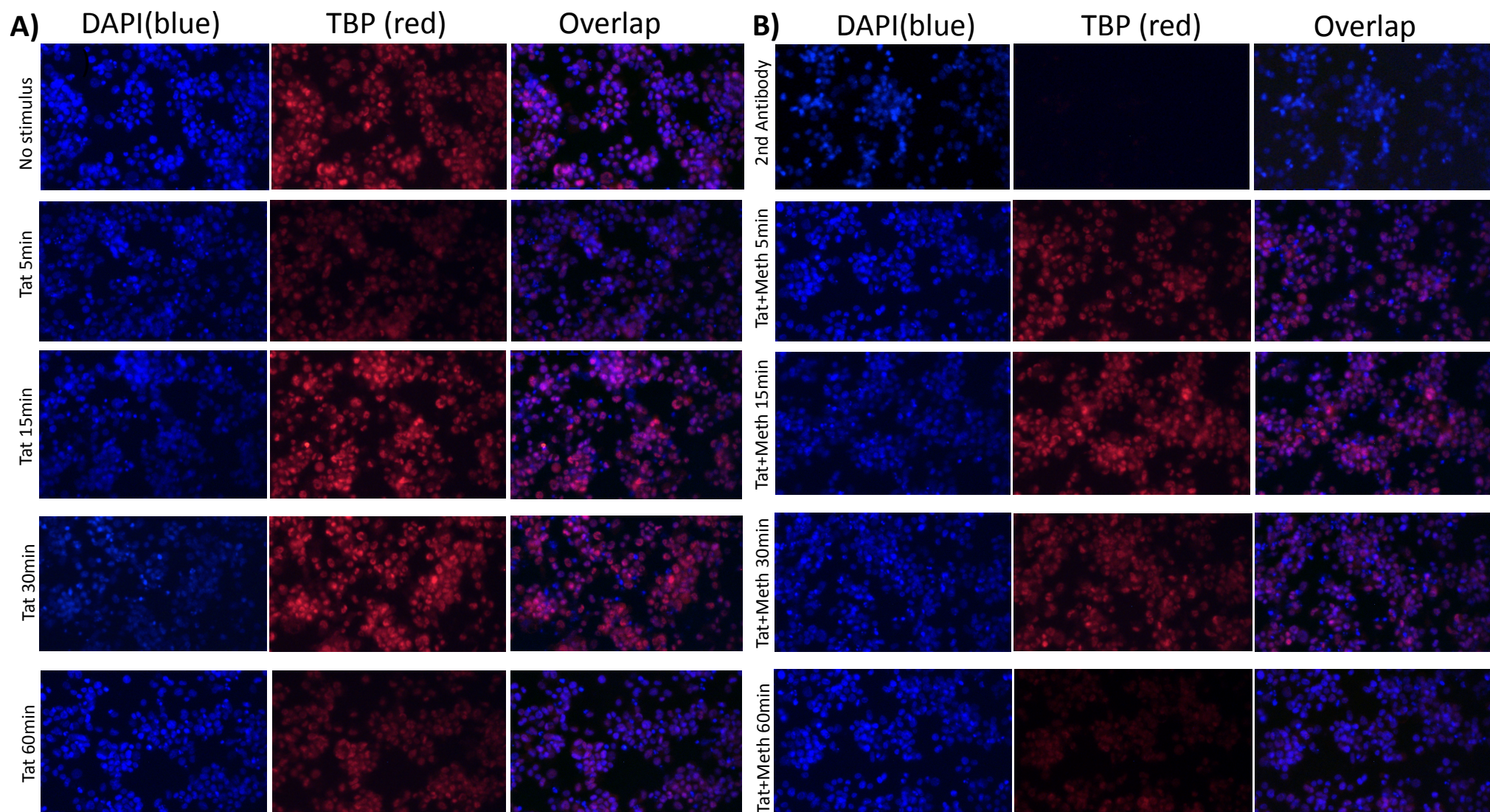

Supplement: Supplementary Figure S1 — TBP nuclear translocation. The PMA-differentiated THP1 cells were stimulated with Meth (60 μM), and/or HIV Tat (10 ng/ml) for examination of TBP nuclear translocation 5, 15, 30, and 60 min following stimulation, by immunocytochemistry. TBP was stained in red, and nuclei were visualized with DAPI (blue). Confocal images were used for the determination of a translocation index, as the percentage of total TBP staining that was co-localized with DAPI. Representative images of (A) Controls and Tat stimulation, (B) Tat+Meth stimulation. This experiment was performed three times in triplicate. [file Data_Sheet_1.PDF]
